# Overexpression of *KCNJ2* enhances maturation of human-induced pluripotent stem cell-derived cardiomyocytes

**DOI:** 10.1186/s13287-023-03312-9

**Published:** 2023-04-15

**Authors:** Jingjun Zhou, Baiping Cui, Xiaochen Wang, Hongkun Wang, Junnan Zheng, Fengfeng Guo, Yaxun Sun, Hangping Fan, Jiaxi Shen, Jun Su, Jue Wang, Haige Zhao, Yiquan Tang, Tingyu Gong, Ning Sun, Ping Liang

**Affiliations:** 1 Key Laboratory of Combined Multi-Organ Transplantation, Ministry of Public Health, The First Affiliated Hospital, Zhejiang University School of Medicine, 79 Qingchun Road, Hangzhou, 310003 Zhejiang China; 2 Institute of Translational Medicine, Zhejiang University, Hangzhou, 310029 Zhejiang China; 3 Institute of Geriatrics (Shanghai University), Affiliated Nantong Hospital of Shanghai University (The Sixth People’s Hospital of Nantong), School of Medicine, Shanghai University, Nantong, 226011 China; 4 Shanghai Engineering Research Center of Organ Repair, School of Medicine, Shanghai University, Shanghai, 200444 China; 5 Department of Cardiovascular Surgery, The First Affiliated Hospital, Zhejiang University School of Medicine, Hangzhou, 310003 Zhejiang China; 6 Department of Cardiology, Sir Run Run Shaw Hospital, Zhejiang University School of Medicine, Hangzhou, 310016 Zhejiang China; 7 State Key Laboratory of Medical Neurobiology and MOE Frontiers Center for Brain Science, Institutes of Brain Science, Fudan University, Shanghai, 200032 China; 8 Wuxi School of Medicine, Jiangnan University, 1800 Lihu Avenue, Wuxi, 214028 Jiangsu China

**Keywords:** iPSC-CMs, *KCNJ2*, Maturation, Electrophysiology, Human-engineered heart tissues

## Abstract

**Background:**

Although human-induced pluripotent stem cell-derived cardiomyocytes (iPSC-CMs) are a promising cell resource for cardiovascular research, these cells exhibit an immature phenotype that hampers their potential applications. The inwardly rectifying potassium channel K_ir_2.1, encoded by the *KCNJ2* gene, has been thought as an important target for promoting electrical maturation of iPSC-CMs. However, a comprehensive characterization of morphological and functional changes in iPSC-CMs overexpressing *KCNJ2* (KCNJ2 OE) is still lacking.

**Methods:**

iPSC-CMs were generated using a 2D in vitro monolayer differentiation protocol. Human KCNJ2 construct with green fluorescent protein (GFP) tag was created and overexpressed in iPSC-CMs via lentiviral transduction. The mixture of iPSC-CMs and mesenchymal cells was cocultured with decellularized natural heart matrix for generation of 3D human engineered heart tissues (EHTs).

**Results:**

We showed that mRNA expression level of *KCNJ2* in iPSC-CMs was dramatically lower than that in human left ventricular tissues. KCNJ2 OE iPSC-CMs yielded significantly increased protein expression of K_ir_2.1 and current density of K_ir_2.1-encoded I_K1_. The larger I_K1_ linked to a quiescent phenotype that required pacing to elicit action potentials in KCNJ2 OE iPSC-CMs, which can be reversed by I_K1_ blocker BaCl_2_. KCNJ2 OE also led to significantly hyperpolarized maximal diastolic potential (MDP), shortened action potential duration (APD) and increased maximal upstroke velocity. The enhanced electrophysiological maturation in KCNJ2 OE iPSC-CMs was accompanied by improvements in Ca^2+^ signaling, mitochondrial energy metabolism and transcriptomic profile. Notably, KCNJ2 OE iPSC-CMs exhibited enlarged cell size and more elongated and stretched shape, indicating a morphological phenotype toward structural maturation. Drug testing using hERG blocker E-4031 revealed that a more stable MDP in KCNJ2 OE iPSC-CMs allowed for obtaining significant drug response of APD prolongation in a concentration-dependent manner. Moreover, KCNJ2 OE iPSC-CMs formed more mature human EHTs with better tissue structure and cell junction.

**Conclusions:**

Overexpression of *KCNJ2* can robustly enhance maturation of iPSC-CMs in electrophysiology, Ca^2+^ signaling, metabolism, transcriptomic profile, cardiomyocyte structure and tissue engineering, thus providing more accurate cellular model for elucidating cellular and molecular mechanisms of cardiovascular diseases, screening drug-induced cardiotoxicity, and developing personalized and precision cardiovascular medicine.

**Supplementary Information:**

The online version contains supplementary material available at 10.1186/s13287-023-03312-9.

## Introduction

The invention of induced pluripotent stem cells (iPSCs) launched a novel era of medical research [1]. The ability to differentiate iPSCs into various cell types provides a numerous and stable source of cells and allows for generation of patient- and disease-specific functional cells to establish human-based disease models [2, 3]. The continuously optimized differentiation and purification approaches allow for obtaining highly purified iPSC-derived cardiomyocytes (iPSC-CMs) efficiently, which offers a human-based, physiology-relevant and scalable cardiomyocyte source [4, 5]. It has been widely demonstrated that iPSC-CMs can be served as a reliable model for elucidating cellular and molecular mechanisms of cardiovascular diseases, screening drug-induced cardiotoxicity, and developing personalized and precision cardiovascular medicine [6–8].

Nevertheless, the application potential of iPSC-CMs is still hampered due to immature phenotypes. iPSC-CMs exhibit spontaneous activities because of relatively depolarized resting membrane potential (RMP), possessing a high risk of arrhythmias. Slower upstroke velocity and a lack or shorter plateau phase of repolarization may exist in the iPSC-CMs attributed to the lower levels of the Na^+^ and L-type Ca^2+^ channels [9, 10]. Under commonly used culture conditions, iPSC-CMs have irregular shapes, flattening and spreading in all directions, whereas adult cardiomyocytes maintain their cylindrical morphology [10, 11]. Moreover, when compared to adult cardiomyocytes, iPSC-CMs rely on glycolysis rather than fatty acid β-oxidation [12]. Consequently, promoting the maturation of iPSC-CMs has become urgent for the cardiovascular applications.

The inwardly rectifying potassium channel K_ir_2.1 encoded by the *KCNJ2* gene plays a major role in the repolarization phase of the cardiac action potential, contributing to stabilization of the RMP [13]. Due to the low current density of K_ir_2.1-encoding I_K1_, iPSC-CMs are prone to exhibit auto-arrhythmic behavior, slower depolarization and impaired sarcoplasmic reticulum (SR)-mediated Ca^2+^ processing [14]. Efforts have been made to produce a more adult-like I_K1_ expression level by intervention in the transcription of *KCNJ2* or increasing the expression of K_ir_2.1, which could generate more stable RMP and mature action potential [15–18]. However, a comprehensive characterization of morphological and functional changes in iPSC-CMs overexpressing *KCNJ2* is still lacking.

## Methods

### Human heart samples

For patients with mitral stenosis or mitral insufficiency who need mitral valve replacement, the diseased mitral lobes and subvalvular structures of the patients, including papillary muscles and tendons, were removed during the operation. The obtained papillary muscles were used as cardiac left ventricular (LV) tissues in this study (Additional file 1: Table 1).

### Culture and maintenance of iPSCs

**Fig. 1:**
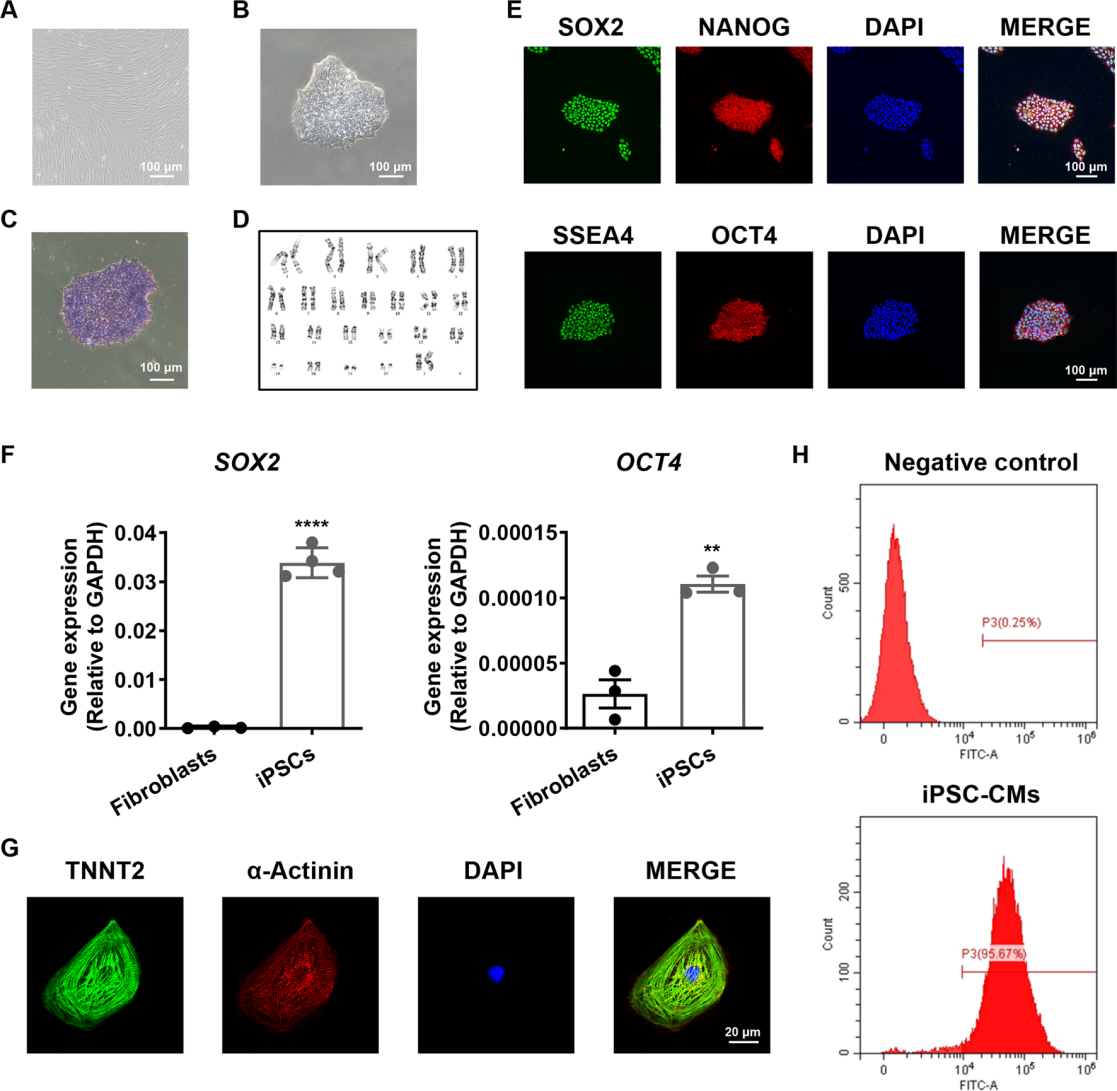
Generation and characterization of iPSC-CMs. **a** Typical morphology of skin fibroblasts. The images were captured by an inverted fluorescence microscope (Nikon ECLIPSE Ti-S). Scale bar, 100 μm. **b** Typical morphology of iPSCs. The images were captured by an inverted fluorescence microscope (Nikon ECLIPSE Ti-S). Scale bar, 100 μm. **c** Representative graph of alkaline phosphatase (ALP) staining of iPSCs. The images were captured by an inverted fluorescence microscope (Nikon ECLIPSE Ti-S). Scale bar, 100 μm. **d** Representative graph of karyotype of iPSCs. **e** Representative graphs of pluripotent staining of iPSCs using SOX2 (green), NANOG (red), SSEA4 (green) and OCT4 (red), DAPI indicates nuclear staining (blue). Fluorescent detection was assessed with a confocal microscope (Nikon A1). Scale bar, 100 μm. **f** Bar graph to compare the mRNA expression of SOX2 and OCT4 by qPCR between skin fibroblasts and iPSCs. *n* = 3–4. **g** Representative graphs of cardiac-specific staining by TNNT2 (green) and α-actinin (red) in iPSC-CMs. DAPI indicates nuclear staining (blue). Fluorescent detection was assessed with a confocal microscope (Nikon A1). Scale bar, 20 μm. **h** Fluorescence-activated cell sorting (FACS) analysis of TNNT2-positive cells in the iPSC-CMs. *n* = 3 independent differentiations

The iPSCs were cultured in feeder-free mTeSR1 (STEMCELL Technologies) on matrigel (Corning)-coated plates at 37 °C with 5% (vol/vol) CO_2_. The media were daily changed. The iPSCs were passaged every 3–4 days using Accutase (STEMCELL Technologies), and resuspended and seeded in mTeSR1 containing 10 μM Y27632 (Selleck). To compare the mRNA expression of *KCNJ2* between human heart tissues and iPSC-CMs, cardiomyocytes were differentiated from 6 iPSC lines, which were derived from 6 different healthy donors (Additional file 1: Table 2 and Figure 1). The iPSC#4 line shown in Additional file 1: Table 2, with a high efficiency of cardiac differentiation, was utilized for downstream investigations in this study.

### Alkaline phosphatase staining

Alkaline phosphatase (ALP) staining was conducted following the manufacturer’s instructions using the VECTOR Blue Alkaline Phosphatase Substrate Kit (Vector Laboratories).

### Cardiac differentiation

The iPSC-CMs were generated using a 2D monolayer differentiation protocol as previously described [19, 20]. Briefly, the iPSCs were dissociated and replated into matrigel-coated 6-well plates. Cells were cultured and expanded to 85% confluence and subsequently treated for 2 days with 6 μM CHIR99021 (Axon Medchem) in RPMI 1640 (Gibco) with B27 supplement minus insulin (Gibco) (RPMI + B27-Insulin) to activate Wnt signaling pathway. On day 2, cells were placed in RPMI + B27-Insulin with CHIR99021 removal. On days 3–4, cells were treated with 5 μM IWR-1 (Millipore) to inhibit Wnt signaling pathway. On days 5–6, cells were removed from IWR-1 treatment and placed in RPMI + B27-Insulin. From day 7 onwards, cells were placed and cultured in RPMI 1640 and B27 supplement with insulin (Gibco) (RPMI + B27 + Insulin) until beating foci were observed. Cells were glucose-starved for 3 days with RPMI + B27 + Insulin for purification. Cardiomyocytes of day 30–40 after cardiac differentiation were utilized for downstream functional assays.

### Fluorescence-activated cell sorting (FACS) analysis of iPSC-CMs

Monolayer iPSC-CMs were dissociated into single cells using 0.25% Tripsin-EDTA (Gibco) for 5 min at 37 °C. Cells were pelleted and fixed with 2% paraformaldehyde (PFA) (Sangon Biotech) for 10 min on ice. Every step was washed with 5% fetal bovine serum (Gibco) in phosphate-buffered saline (PBS) (Sangon Biotech) before sample centrifugation. Cells were stained with TNNT2 (Abcam) at 4 °C. FITC-conjugated goat anti-mouse IgG antibody (Invitrogen) was used as the secondary antibody.

### Overexpression of *KCNJ2* in iPSC-CMs

The *KCNJ2* fragment was digested with NheI and BamHI endonucleases (NEB) from the KCNJ2-green fluorescent protein (GFP)-IRES plasmid. The mixture was subsequently separated by a 1% agarose gel and purified by TIANgel Midi Purification Kit (TIANGEN). The purified product was then subcloned into the GFP-carrying vector (pCDH-CMV-MCS-EF1-copGFP). The recombinant sequence was confirmed by Sanger sequencing. The recombinant lentiviral plasmid and the lentivirus packaging plasmid were co-transfected into human embryonic kidney 293 T (HEK293T) cells with Lipofectamine 3000 (Invitrogen), and virus were harvested 3–4 days after the transfection. The harvested virus was filtered using a 0.45 μm cell strainer (Millipore), and concentrated by 5 × PEG8000 (Biosharp). The iPSC-CMs were grown in 12-well plate to 80% confluence, and then transfected with purified virus according to the virus titer. Polybrene (5–10 μg/ml) (Sigma-Aldrich) was added in parallel to enhance the efficiency of transfection. 3 days after the transfection, the images were collected using an inverted confocal microscope (Nikon) and NIS-Elements AR software (Nikon). The multiplicity of infection (MOI) used for transducing the iPSC-CMs is 12 in our study. Successfully transfected cells exhibiting green fluorescence in each well reached 85% or more.

### Immunofluorescent staining

Cells were fixed with 4% PFA (Sangon Biotech) for 15 min, permeabilized with 0.2% Triton X-100 (Sangon Biotech) for 5 min, followed by blocking with 3% BSA (Sigma-Aldrich) for 1 h. Cells were subsequently stained with appropriate primary antibodies and AlexaFluor conjugated secondary antibodies (Life Technologies). Nuclei were stained with DAPI (Roche Diagnostics). Every step was washed with PBS (Sangon Biotech) for 5 min. For the staining of pluripotency markers, the primary antibodies were OCT4 (Santa Cruz Biotechnology), NANOG (Santa Cruz Biotechnology), SSEA-4 (Abcam) and SOX2 (Abcam). For the staining of cardiac-specific markers, the primary antibodies were TNNT2 (Abcam) and α-actinin (Abcam). Pictures were taken with 20 × or 60 × objective on confocal microscope (Nikon, A1) using NIS-Elements AR software (Nikon). During the experiments, the same imaging threshold and exposure time were applied to maintain the consistency of image analysis. The area, perimeter, circularity and elongation of cells were counted by the Image J software.

Three-dimensional (3D) human engineered heart tissues (EHTs), ECM, and ECM seeded with cells were fixed in 4% PFA, processed for embedding (OCT, Sakura Finetek), and then sectioned into 8 μm-thick sections. The frozen slides were permeabilized with 0.5% Triton X-100 for 15 min and blocked with normal goat serum for 30 min. Samples were then incubated with the following antibodies diluted in 3% BSA blocking solution and 1% goat serum for 12 h at 4 °C: Laminin (Thermo Fisher Scientific), fibronectin (Abcam), collagen III (Abcam), TNNT2 (Abcam), α-SMA (Abcam), Von Willebrand Factor (VWF) (Abcam) and connexin 43 (CX43) (Abcam). The next day, after three 5-min washes with PBS, samples were stained at room temperature for 1 h with fluorescent secondary antibodies (Abcam) followed by 10 min of DAPI staining for nucleus visualization. Fluorescent detection was assessed with a fluorescence microscope Leica DMi8 (Leica) or confocal microscope (Leica).

### Patch clamp recordings

The iPSC-CMs were dissociated to obtain single cells, which were seeded on matrigel-coated glass coverslips (Warner Instruments). Action potentials were recorded from iPSC-CMs using an EPC-10 patch clamp amplifier (HEKA). A rapid solution exchanger (Bio-logic Science Instruments) was applied to achieve continuous extracellular solution. All signals were acquired using PatchMaster software (HEKA), and filtered at 1 kHz and digitized at 10 kHz. Data analyses were performed using Igor Pro (Wavemetrics) and GraphPad Prism (GraphPad Software). A TC-344C dual channel heating system (Warner Instruments) was used to maintain the temperature at 35.5–37 °C. Tyrode’s solution was used as the external solution. The internal solution contained 140 mM KCl, 5.0 mM NaCl, 10 mM HEPES, 5 mM Mg-ATP and 5 mM EGTA (pH 7.2 with KOH). In quiescent iPSC-CMs, action potentials were elicited by pulse stimulations (500–800 pA for 5 ms) with a pacing frequency at 1 Hz. Key action potential parameters were quantified, including maximal diastolic potential (MDP), overshoot, action potential amplitude (APA), action potential duration at 50% and 90% repolarization (APD_50_ and APD_90_), maximal upstroke velocity (V_max_) and beating rate. To isolate I_K1_ from iPSC-CMs, current was elicited by 300 ms voltage pulses from − 120 to + 20 mV in 10 mV increments at a holding potential of − 40 mV. 1 μM nifedipine and 10 μM tetrodotoxin (TTX) were used to block the Ca^2+^ channel and Na^+^ channel, respectively.

### Ca^2+^ imaging

The iPSC-CMs grown on coverslips were loaded with RPMI 1640 medium without phenol red (Gibco) supplemented with 5 μM Fura-2 AM (Invitrogen) for 30 min in the dark at room temperature. After washing with pre-warmed DPBS and RPMI 1640 for 2 times, iPSC-CMs were immersed in imaging buffer for 30 min before imaging experiments. For imaging, iPSC-CMs were placed in a chamber equipped with a temperature-controller under constant perfusion of 37 °C imaging buffer. Ca^2+^ signaling was made by recording the fluorescence of cells using an Ultra High Speed Wavelength Switcher (Lambda DG-4, Sutter Instruments) with a CCD camera (Zyla, Andor) mounted on an inverted microscope (Eclipse Ti, Nikon). In quiescent iPSC-CMs, fluorescent signals were obtained upon excitation at 340 nm (F_340_) and 380 nm (F_380_) with field stimulation at a rate of 0.5 Hz. Amplitude of Ca^2+^ transient is defined as the ratio of F_340_/F_380_.

### RNA sequencing

The sequencing data was filtered with SOAPnuke (v1.5.2) by (1) removing reads containing sequencing adapter; (2) removing reads whose low-quality base ratio (base quality less than or equal to 5) is more than 20%; (3) removing reads whose unknown base ('N' base) ratio is more than 5%, afterward clean reads were obtained and stored in FASTQ format. The clean reads were mapped to the reference genome using HISAT2 (v2.0.4). Bowtie2 (v2.2.5) was applied to align the clean reads to the reference coding gene set; then expression level of gene was calculated by RSEM (v1.2.12). The heatmap was drawn by pheatmap (v1.0.8) according to the gene expression in different samples. Essentially, differential expression analysis was performed using the DESeq2(v1.4.5) with Q value ≤ 0.05. To take insight into the change of phenotype, GO (http://www.geneontology.org/) and KEGG (https://www.kegg.jp/) enrichment analysis of annotated different expressed gene was performed by Phyper (https://en.wikipedia.org/wiki/Hypergeometric_distribution) based on hypergeometric test. The significant levels of terms and pathways were corrected by Q value with a rigorous threshold (Q value ≤ 0.05) by Bonferroni.

### Real-time quantitative PCR (qPCR)

The iPSC-CMs were lysed using Trizol (Invitrogen) followed by RNA extraction. RNA concentration was measured using UV spectrophotometry at 260 nm (Nanodrop 2000, Thermo Scientific). cDNA was obtained using the High Capacity cDNA Reverse transcription Kit (Applied Biosystems). SYBR Green PCR Master Mix (Takara) was used for qPCR. Primer sequences used in this study were listed in Additional file 1: Table 3. Each reaction was run in triplicates using an Applied Biosystems Viia7 Dx (Thermo Fisher Scientific). The average expression of the housekeeping gene *GAPDH* was used as a reference for standardized gene expression.

### Western blot

The iPSC-CMs were grown in 6-well plates to 80% confluence, detached with TrypLE (Gibco), and then pelleted at 10,000 rpm for 3–5 min at 4 °C. The pellets were washed twice with Dulbecco’s Phosphate-Buffered Saline (DPBS) (Gibco) and then lysed in RIPA (Solarbio). Lysates were placed on ice for 30 min and subsequently centrifuged at 12,000 rpm for 15 min to collect the supernatants. Protein concentration was measured using a BCA kit (Thermo Fisher Scientific). Protein was separated in 12% protein precast gels (Genscript) and transferred to a PVDF membrane. Block the membrane in 5% skimmed milk in Tris-Buffered Saline and Tween 20 (TBST) for 1 h, incubate overnight in 4 °C with primary antibodies, and then incubate with secondary antibodies (Cell Signaling Technology) at room temperature for 1.5 h. Primary antibodies include K_ir_2.1 (Proteintech), Na_v_1.5 (Alomone Labs), RYR2 (Abcam), TNNT2 (Abcam), MYBPC3 (Santa Cruz Biotechnology), KCNH2 (Santa Cruz Biotechnology), Ca_v_1.2 (Abcam) and GAPDH (Abmart).

### Measurements of oxygen consumption

Seahorse XFe96 Analyzer with XF Cell Mito Stress Test Kit (Agilent Technologies) was applied for measuring the mitochondrial respiration in iPSC-CMs following the manufacturer's instructions. Briefly, iPSC-CMs were seeded at a density of 4 × 10^4^ cells per well on Seahorse XF96 cell culture microplates (Agilent Technologies) for 7 days. After three baseline measurements, oligomycin (1.5 μM), carbonyl cyanide p-trifluoromethoxyphenylhydrazone (FCCP, 4 μM), and rotenone/antimycin A (1 μM) were sequentially added to each well.

### Human umbilical cord-derived mesenchymal stem cell isolation and culture

Fresh umbilical cords were collected and processed within the optimal period of 6 h as previously described [21]. Human umbilical cord-derived mesenchymal stem cell (hUC-MSC) isolation was performed in the current Good Manufacturing Practice (cGMP)-accredited laboratory. Firstly, umbilical cords were rinsed twice in PBS containing 5% penicillin and 5% streptomycin to remove the cord blood. Then umbilical cords were cut into pieces of 1–3 mm^3^ after the removal of umbilical cord vessels and cultured in mesenchymal stem cells basic medium (Beijing Yocon Biology Co., Ltd.) supplemented with a serum-free replacement. After initial plating, the medium was changed every 3 days. Well-developed colonies could be observed when fibroblast-like cells reached 80% confluence, which were trypsinized with 0.25% trypsin–EDTA (Invitrogen) and then passaged into new flasks for further expansion. The expression of series of cell surface markers was used to characterize the hUC-MSCs by flow cytometry with positive cell surface markers (CD90, CD105, CD73 and CD44) and negative surface markers (CD34, CD45 and HLA-DR).

### Animals

Sprague Dawley (SD) rats were purchased from Shanghai Slake Laboratory Animal Co. LTD. All rats were raised in the specific pathogen free (SPF) level animal room maintained at 25 °C, light and dark alternately for 12 h and food and water available ad libitum. A total of 12 male SD rats weighted 200–300 g were used in this study. Two rat hearts were used for heart tissue sectioning, and the rest rat hearts were used for preparing decellularized natural heart extracellular matrix (ECM). All animal experiment protocols were approved by the Institutional Animal Care and Use Committee (IACUC) of Fudan University. To harvest rat hearts for ex vivo experiments, rats were euthanized by CO_2_ inhalation in the home cage with carbon dioxide infusion at 50% VDR/min.

### Decellularization of isolated rat heart and generation of 3D human EHTs

To prepare decellularized natural heart ECM, the heart isolated from the 12-week-old SD rat was decellularized through retrograding coronary perfusion referring to our previously published protocol [22]. Briefly, harvest the hearts immediately after euthanasia of adult rats and then a blunted 20-gauge needle was cannulated into the ascending aorta following fix the heart to the perfusion device. Flush the blood of heart vessels with ~ 500 ml sterile deionized water perfusion for 30 min at the rate of 2.0 ml/min firstly, followed decellularization by perfusion with 1% sodium dodecyl sulfate (SDS) for 2 h, 1% Triton X-100 with 0.5% EDTA (pH 8.0) for another 30 min successively at room temperature. Then the heart was washed with antibiotic-containing deionized water and PBS (100 U/ml penicillin (Life Technologies), 100 mg/ml streptomycin (Life Technologies) and 1.25 mg/ml amphotericin B (Sigma-Aldrich)) for 2 h.

The decellularized heart ECM was cut into pieces of 0.5 cm × 0.5 cm size using a sterile surgical scissor in a biosafety cabinet. Individual ECM pieces were put in 48-well plates as a sheet, with endocardium side up. The iPSC-CMs and human mesenchymal stem cells (MSCs) were mixed and seeded in the ratio of 3:1 into the ECM at a concentration of 5 × 10^5^ mm^3^, and this cellular heterogeneity facilitated electrical conduction [23]. The 3D human EHTs were then cultured in DMEM with 10% FBS for another 7 days.

### Compounds and solutions

All the chemicals used in the electrophysiological experiments were purchased from Sigma-Aldrich. Fura-2 AM was purchased from Invitrogen and stock solutions were both prepared in 1 mM in 20% Pluronic F-127 (Sangon Biotech) dissolved in DMSO (Sigma-Aldrich). Isoproterenol was purchased from Sigma-Aldrich and stock solutions were prepared as 1 mM in H_2_O. E-4031 was purchased from Sigma-Aldrich and stock solutions were prepared as 10 mM in H_2_O.

### Statistical analysis

Unpaired two-tailed Student’s t test was applied to compare the statistical significance and one-way ANOVA was applied to compare multiple groups. A *p* value of < 0.05 was evaluated statistically significant. Data were shown as mean ± SEM and analyzed by GraphPad Prism (GraphPad Software).

## Results

### Generation and characterization of iPSC-CMs

The previously established iPSC line was utilized in this study [24], which was derived from skin fibroblasts of a healthy control subject (Fig. 1a). The iPSCs exhibited a human embryonic stem cell-like morphology (Fig. 1b) and ALP staining (Fig. 1c), maintained normal karyotype profile (Fig. 1d), stained positive for pluripotent markers (SOX2, NANOG, SSEA4 and OCT4) (Fig. 1e), and expressed pluripotency genes (*SOX2* and *OCT4*) (Fig. 1f). Using a 2D in vitro monolayer differentiation protocol, we successfully differentiated iPSCs into cardiomyocytes. Spontaneously beating foci at around day 8 after induction of cardiac differentiation were clearly observed. The generated monolayer iPSC-CMs were mechanically and enzymatically dissociated into single cells, which exhibited positive staining of cardiac markers TNNT2 and α-actinin (Fig. 1g). FACS analysis revealed > 95% of TNNT2-positive cells, indicating a high yield of cardiomyocytes (Fig. 1h).

### Overexpression of *KCNJ2* gives rise to enhanced I_K1_ in iPSC-CMs

**Fig. 2:**
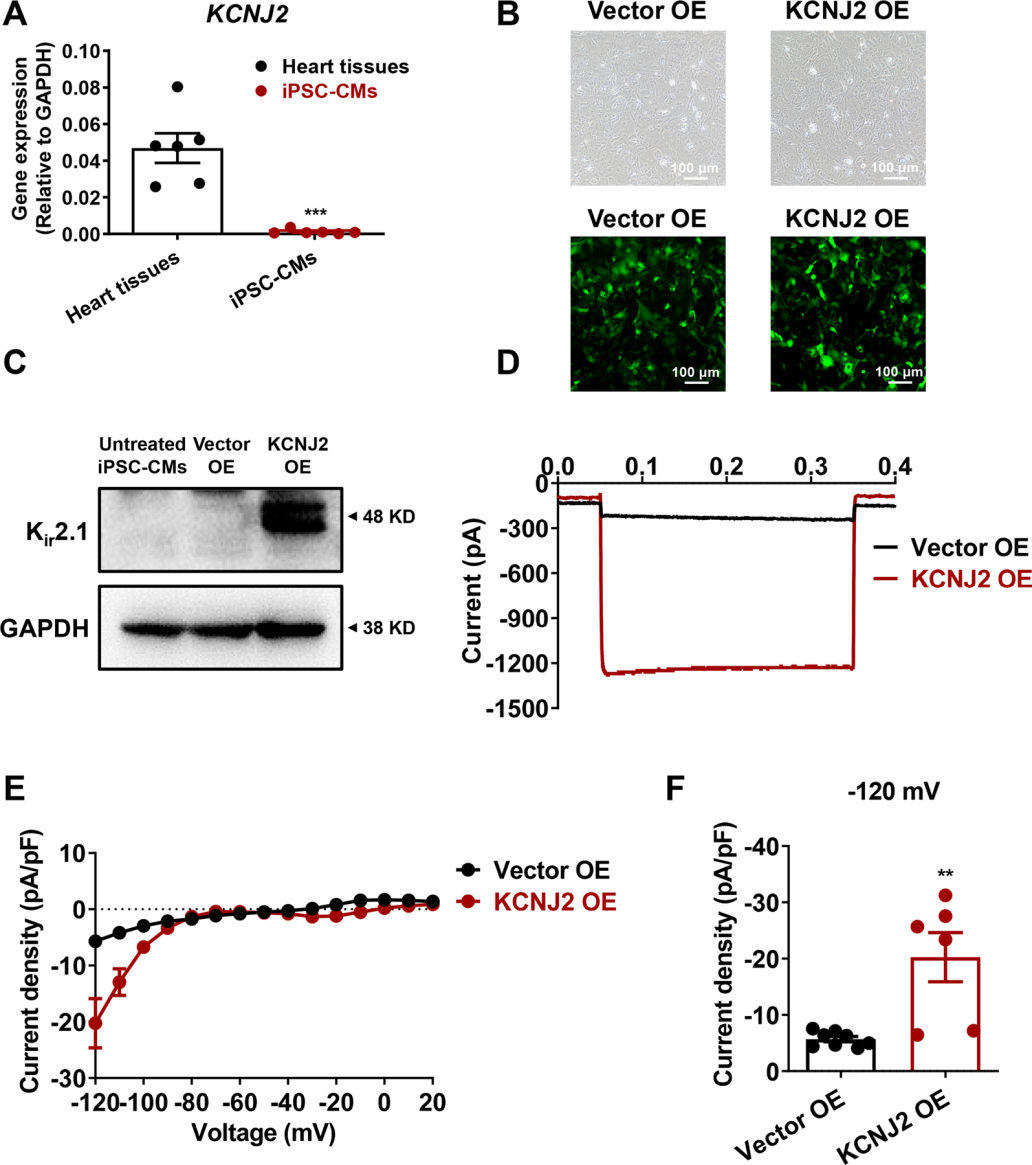
Overexpression of KCNJ2 in iPSC-CMs gives rise to enhanced IK1. **a** Bar graph to compare the mRNA expression of KCNJ2 between human heart tissues and iPSC-CMs. *n* = 6 different human heart tissues or different iPSC lines. **b** iPSC-CMs were transduced with GFP only (negative control, Vector OE) or Kir2.1 protein labeled with GFP (KCNJ2-GFP, KCNJ2 OE) using the lentiviral transfection system. 72 h after transfection, the representative graphs were captured to reveal the high level of overexpression. GFP indicates successfully transfected cells. The images were captured by an inverted fluorescence microscope (Nikon ECLIPSE Ti-S). Scale bar, 100 μm. **c** Kir2.1 protein abundance was evaluated among untreated, Vector OE and KCNJ2 OE iPSC-CMs by Western blot analysis. Full-length blots are presented in Additional file 1: Figure 2. **d** Representative current tracings of IK1 recorded from Vector OE and KCNJ2 OE iPSC-CMs by single-cell patch clamp at − 120 mV. **e** I–V plots of IK1 of Vector OE and KCNJ2 OE iPSC-CMs by single-cell patch clamp recordings from − 120 to + 20 mV. **f** Bar graph to compare the current density of IK1 between Vector OE (*n* = 8 cells) and KCNJ2 OE (*n* = 6 cells) iPSC-CMs at − 120 mV

To determine the expression level of *KCNJ2* in iPSC-CMs, we performed qPCR experiments to compare the mRNA-level expression of *KCNJ2* between iPSC-CMs and adult cardiac LV tissues. We observed that the mRNA expression level of *KCNJ2* in cardiomyocytes derived from 6 different iPSC lines was dramatically lower than that in 6 different LV tissues (Fig. 2a). This observation led us to overexpress the *KCNJ2* gene in iPSC-CMs. Human *KCNJ2* construct with GFP tag was created, and either GFP only (negative control) or *KCNJ2*-GFP was overexpressed in iPSC-CMs via lentiviral transduction. After 72 h of transfection, strong green fluorescence can be detected in more than 85% of the transfected iPSC-CMs, indicating that a high-efficiency overexpression system has been successfully established (Fig. 2b). Western blot analysis demonstrated a significantly increased protein expression of K_ir_2.1 in iPSC-CMs overexpressing *KCNJ2* (KCNJ2 OE iPSC-CMs), when compared to untreated basal iPSC-CMs and iPSC-CMs overexpressing vector only (Vector OE iPSC-CMs) (Fig. 2c and Additional file 1: Figure 2). To note, single-cell voltage clamp recordings revealed that the current density of K_ir_2.1-encoded I_K1_ was markedly larger in KCNJ2 OE iPSC-CMs as compared to their Vector OE counterparts (Vector OE: − 5.70 ± 0.47 pA/pF; KCNJ2 OE: − 20.25 ± 4.38 pA/pF) (Fig. 2d–f). These results indicate that overexpression of *KCNJ2* in iPSC-CMs gives rise to enhanced I_K1_.

### Overexpression of *KCNJ2* enhances electrophysiological maturation in iPSC-CMs

**Fig. 3:**
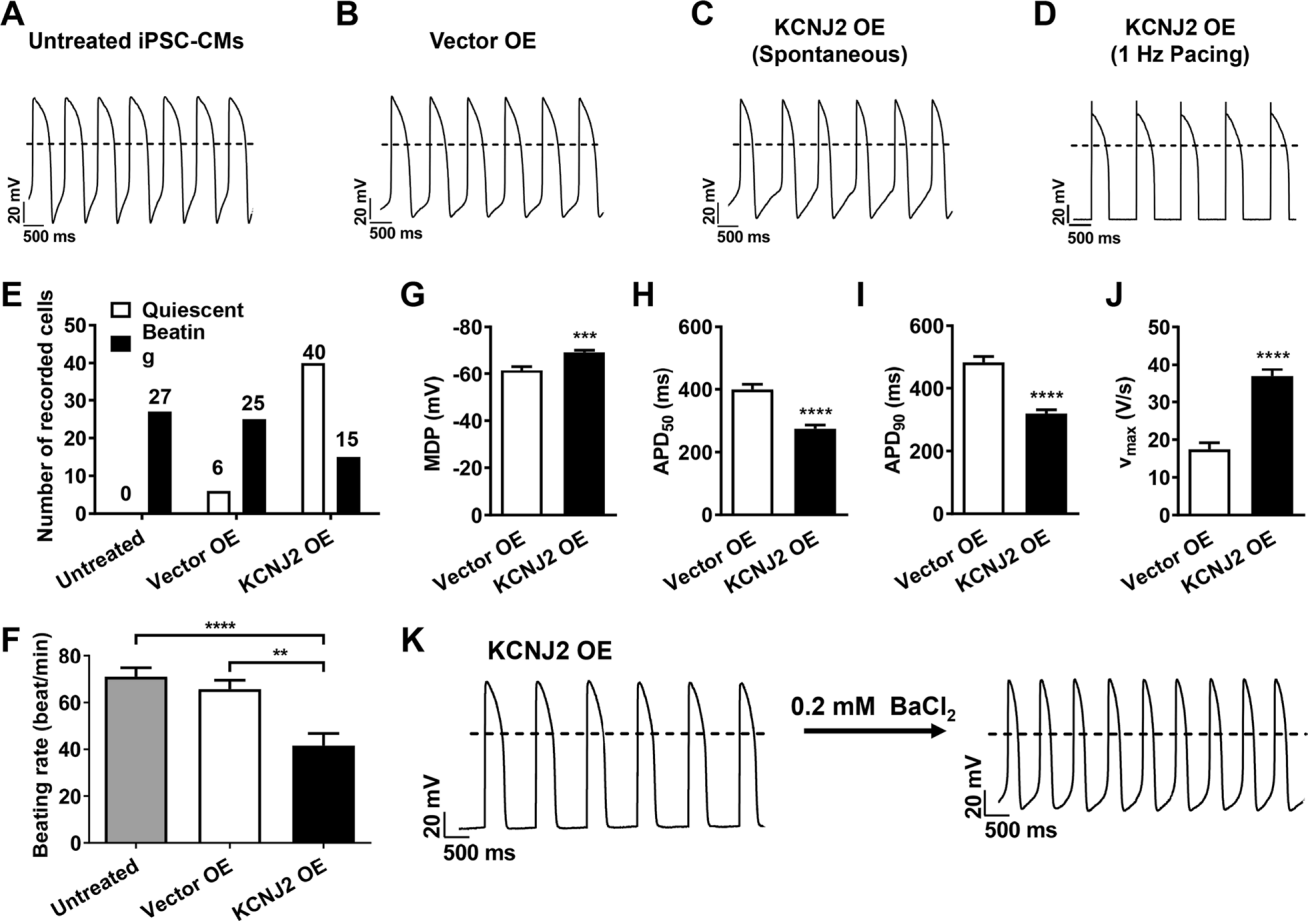
Overexpression of KCNJ2 enhances electrophysiological maturation in iPSC-CMs. **a–d** Representative action potential tracings recorded by single-cell patch clamp from untreated, Vector OE and KCNJ2 OE iPSC-CMs, respectively. Dash lines indicate 0 mV. **e** Bar graph to compare the number of quiescent and spontaneous beating cells in untreated, Vector OE and KCNJ2 OE iPSC-CMs, respectively. **f** Bar graph to compare the beating rate in untreated (*n* = 27 cells), Vector OE (*n* = 25 cells) and KCNJ2 OE (*n* = 15 cells) iPSC-CMs, respectively. **g–j** Bar graphs to compare MDP, APD50, APD90 and Vmax between Vector OE (*n* = 13 cells) and KCNJ2 OE (*n* = 40 cells) iPSC-CMs. **k** Representative action potential tracings recorded by single-cell patch clamp from KCNJ2 OE iPSC-CMs before and after 0.2 mM BaCl2 (IK1 blocker) treatment

To investigate if overexpression of *KCNJ2* in iPSC-CMs gives rise to functional consequences, we next performed current clamp to record action potentials from basal, Vector OE, and KCNJ2 OE iPSC-CMs (Fig. 3a–d). We observed that similar with untreated basal iPSC-CMs (quiescent: 0%; beating: 100%), the majority of Vector OE iPSC-CMs showed a spontaneous beating profile (quiescent: 19%; beating: 81%) (Fig. 3e). In contrast, the majority of KCNJ2 OE iPSC-CMs were quiescent that required pacing to elicit action potentials (quiescent: 73%; beating: 27%) (Fig. 3e). Beating rate was also calculated in iPSC-CMs with spontaneous beating, and KCNJ2 OE iPSC-CMs showed a significantly slower beating rate, when compared to untreated basal or Vector OE iPSC-CMs (untreated basal: 71.00 ± 3.82 beats/min; Vector OE: 65.73 ± 3.79 beats/min; KCNJ2 OE: 41.53 ± 5.24 beats/min) (Fig. 3f). In addition, we observed prominent changes of key action potential parameters in KCNJ2 OE iPSC-CMs as compared to their Vector OE counterparts, including hyperpolarized MDP, shortened APD_50_ and APD_90_, and increased maximal upstroke velocity (V_max_) (Fig. 3g–j). Notably, pharmacological inhibition of I_K1_ by 0.2 mM BaCl_2_ in KCNJ2 OE iPSC-CMs recovered spontaneous beating (6 out of 10 recorded cells) and restored the action potential profile observed in untreated basal or Vector OE iPSC-CMs (Fig. 3k).

### Overexpression of *KCNJ2* enhances Ca^2+^ signaling maturation in iPSC-CMs

**Fig. 4:**
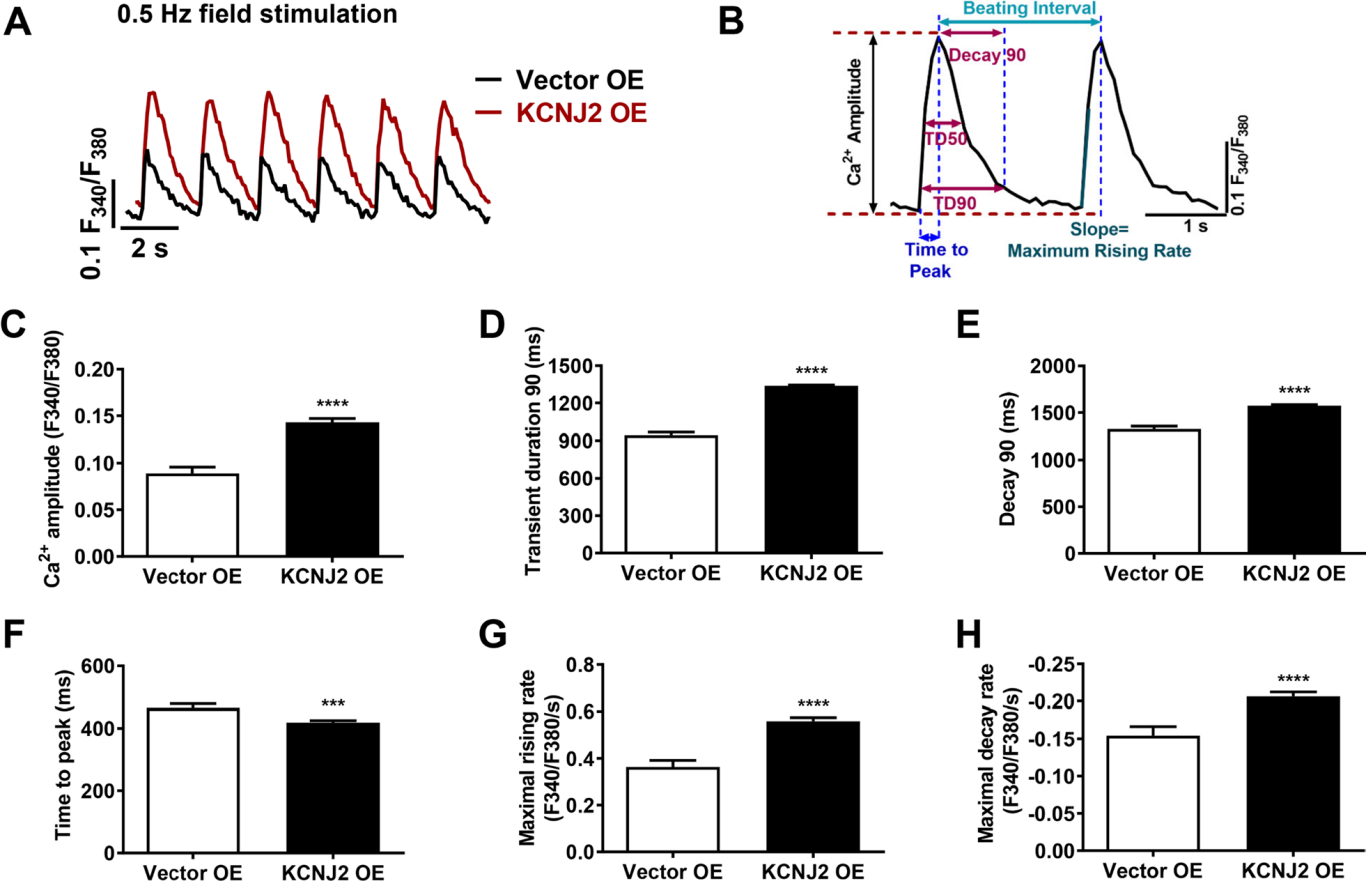
Overexpression of KCNJ2 enhances Ca2+ signaling maturation in iPSC-CMs. **a** Representative Ca^2+^ transient tracings recorded by Fura-2 Ca2+ imaging from Vector OE (black) and KCNJ2 OE (red) iPSC-CMs under 0.5 Hz field stimulation. **b** Schematic diagram of Ca2+ transients demonstrating key parameters to analyze. Transient duration 90 denotes the full duration of a Ca^2+^ transient at 10% of maximal amplitude. Decay 90 denotes the difference in time from the peak of Ca^2+^ transient to the point when intracellular Ca^2+^ returns 90% from the maximal amplitude. **c–h** Scheme to bar graphs to compare key Ca^2+^ transient parameters between Vector OE (*n* = 34 cells) and KCNJ2 OE (*n* = 66 cells) iPSC-CMs, including transient amplitude, transient duration 90, decay 90, time to peak, maximal rising rate, and maximal decay rate

Ca^2+^ transients were recorded from iPSC-CMs under 0.5 Hz stimulation by Fura-2 AM-based Ca^2+^ imaging assay and key parameters were calculated and compared (Fig. 4a, b). We observed that the transient amplitude was significantly increased in KCNJ2 OE iPSC-CMs, when compared to Vector OE iPSC-CMs (Fig. 4c). We also observed markedly prolonged transient 90 duration and decay 90, but abbreviated time to peak in KCNJ2 OE iPSC-CMs (Fig. 4d–f). Moreover, the maximal rising and decay rates were both significantly increased in KCNJ2 OE iPSC-CMs (Fig. 4g, h).

### Overexpression of *KCNJ2* enables more accurate prediction of drug-induced cardiotoxicity in iPSC-CMs

**Fig. 5:**
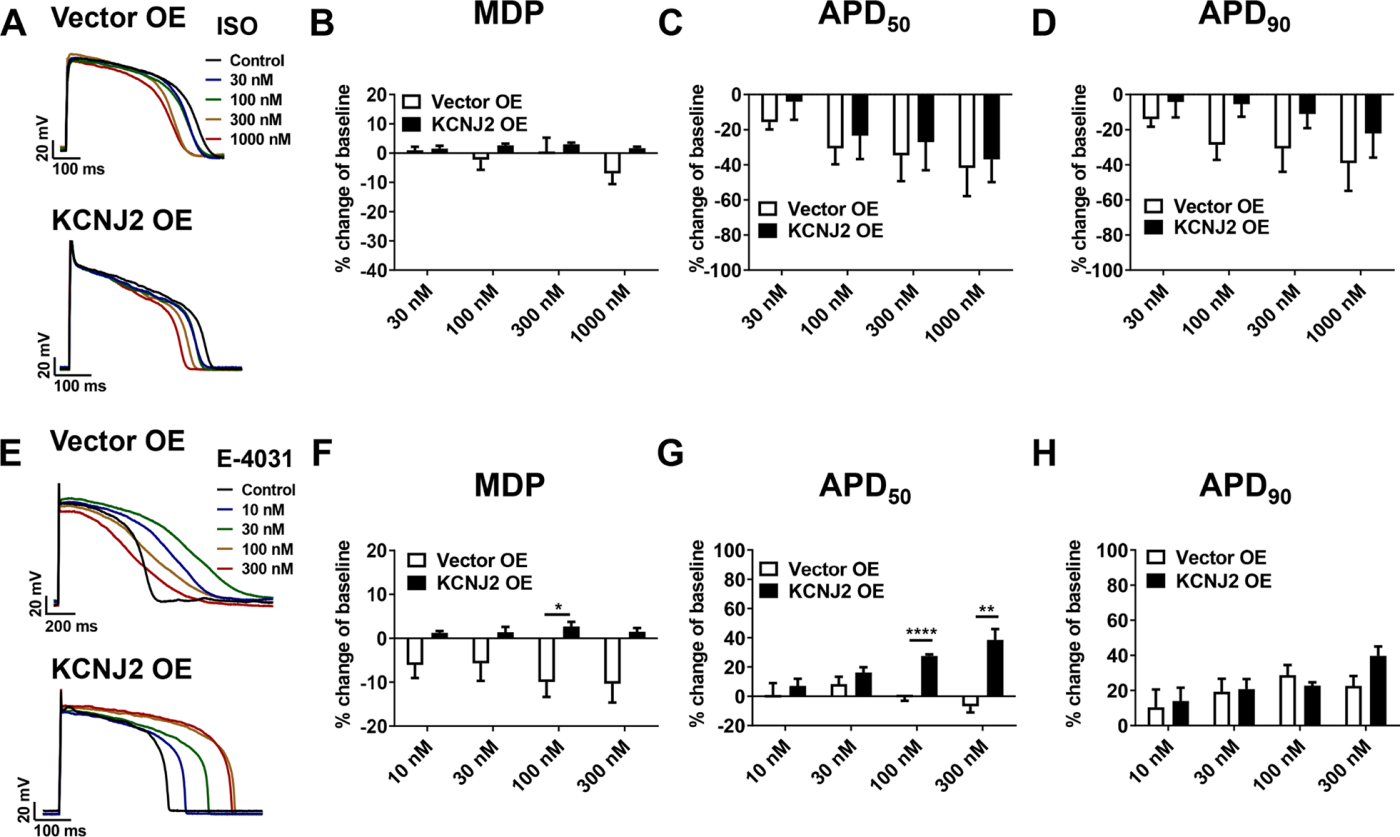
Overexpression of KCNJ2 leads to more stable drug-induced responses in iPSC-CMs. **a** Representative action potential tracings recorded from Vector OE and KCNJ2 OE iPSC-CMs with acute treatment of 0, 30, 100, 300 and 1000 nM ISO, respectively. **b–d** Acute effects of ISO on MDP, APD50 and APD90 in Vector OE (*n* = 5 cells) and KCNJ2 OE (*n* = 6 cells) iPSC-CMs, respectively. **e** Representative action potential tracings recorded from Vector OE and KCNJ2 OE iPSC-CMs with acute treatment of 0, 10, 30, 100 and 300 nM E-4031 (hERG-specific blocker), respectively. **f–h** Acute effects of E-4031 on MDP, APD50 and APD90 in Vector OE (*n* = 5 cells) and KCNJ2 OE (*n* = 5 cells) iPSC-CMs, respectively

It has been reported that low level of I_K1_ may contribute to immature action potential that might bias drug-induced responses [14]. we next investigated if overexpression of *KCNJ2* may change the drug response profile in iPSC-CMs. We first assessed the effects of β-adrenergic agonist Isoproterenol (ISO) on single-cell action potentials by patch clamp recordings from Vector OE and KCNJ2 OE iPSC-CMs under 0.5 Hz pacing (Fig. 5a). We observed that ISO had minimal effect on MDP within a wide range of concentrations (30–1000 nM) in both groups (Fig. 5b). ISO-induced concentration-dependent APD_50_ and APD_90_ shortening was similarly observed in the two groups (Fig. 5c, d), which was consistent with the previous study [25]. We also assessed the effects of E-4031, a specific hERG blocker, on action potentials from Vector OE and KCNJ2 OE iPSC-CMs (Fig. 5e). In Vector OE iPSC-CMs, treatment of E-4031 depolarized MDP in a concentration-dependent manner, suggesting that I_Kr_ has a significant contribution to the maintenance of the MDP (Fig. 5f). High-concentration treatment of E-4031 failed to produce APD prolongation in a concentration-dependent manner, which may be related to the depolarizing effect of the drug (Fig. 5g, h). In contrast, when treated on KCNJ2 OE iPSC-CMs, E-4031 had minimal effect on MDP and significantly prolonged APD in a concentration-dependent manner (Fig. 5f–h).

### RNA sequencing reveals differential transcriptomic profile in KCNJ2 OE iPSC-CMs

**Fig. 6:**
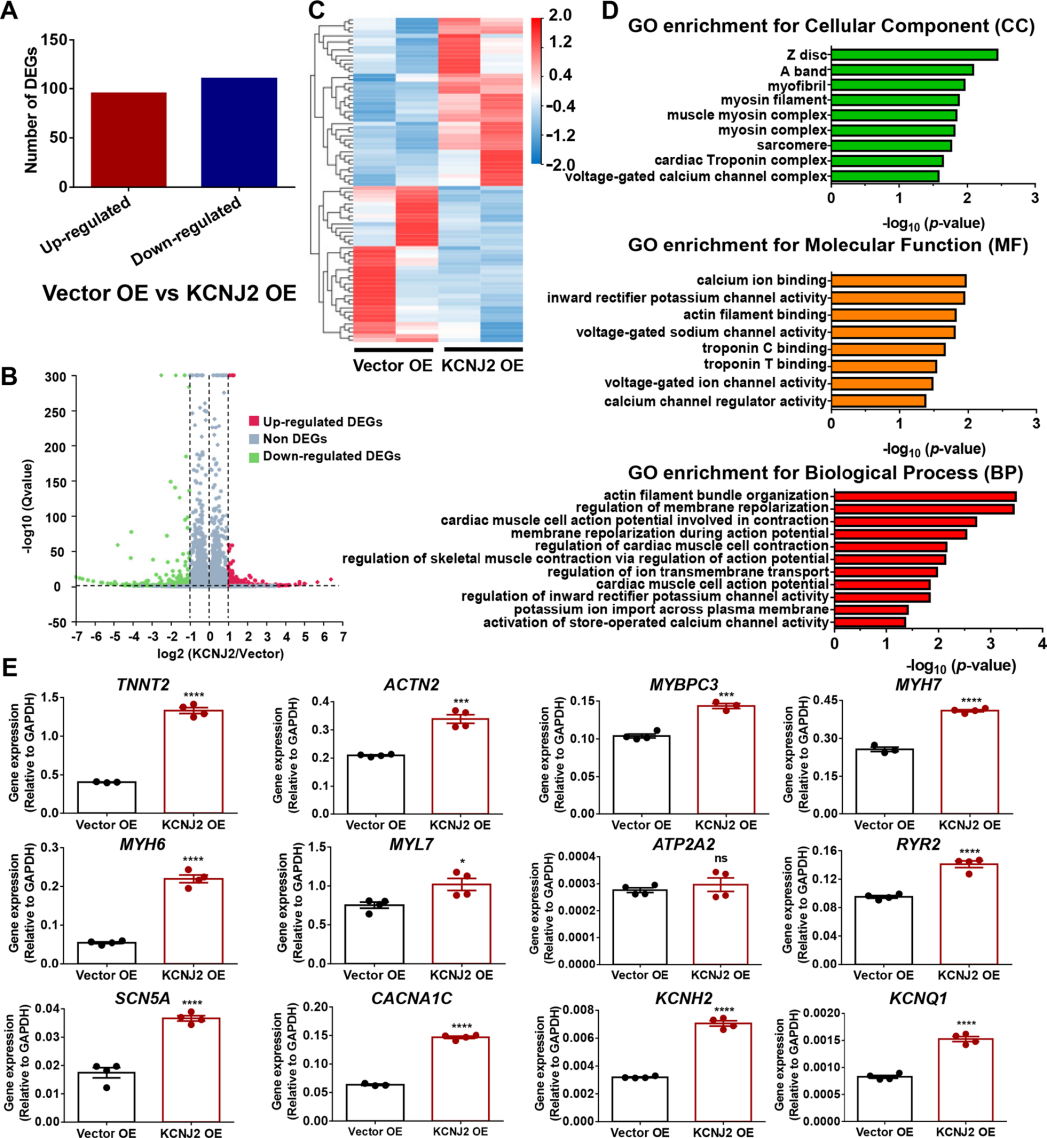
RNA sequencing reveals distinct transcriptomic profile in KCNJ2 OE iPSC-CMs. **a** Bar graph showing the number of up- and downregulated differentially expressed genes (DEGs) between Vector OE and KCNJ2 OE iPSC-CMs. **b** Volcano plot showing the total of 205 DEGs. Red dots represent 95 upregulated DEGs. Green dots represent 110 downregulated DEGs. Gray dots represent non-DEGs. **c** Heat map demonstrating the differential gene expression pattern between Vector OE and KCNJ2 OE iPSC-CMs. **d** Top enriched Gene ontology (GO) analysis for cellular compartment (CC), molecular function (MF) and biological process (BP), respectively. **e** qPCR validation of a panel of genes associated with cardiac sarcomere proteins (TNNT2, ACTN2, MYBPC3, MYH7, MYH6 and MYL7), Ca^2+^ handing proteins (ATP2A2 and RYR2), and ion channels (SCN5A, CACNA1C, KCNH2 and KCNQ1). *n* = 3–4 independent differentiations

Taking a step forward, we sought to investigate whether *KCNJ2* overexpression may lead to change of transcriptomic profile in iPSC-CMs. Genome-wide RNA sequencing (RNA-Seq) were performed by comparing Vector OE and KCNJ2 OE iPSC-CMs. We observed that 205 genes out of 18,236 total genes were differentially expressed in KCNJ2 OE iPSC-CMs versus Vector OE iPSC-CMs, of which 95 were upregulated and 110 were downregulated (Fig. 6a–c). Gene ontology (GO) analysis demonstrated that differentially expressing genes (DEGs) were not only enriched in terms related to ion channels and electrophysiology such as “calcium ion binding”, “inward rectifier potassium channel activity”, “regulation of membrane repolarization” and “cardiac muscle cell action potential involved in contraction”, but also enriched in terms related to cardiac structure such as “Z disc”, “myosin filament”, “troponin C binding”, “troponin T binding” and “actin filament bundle organization” (Fig. 6d). A panel of 12 genes related to ion channels, Ca^2+^ handling, or cardiac sarcomere were selected for validation by qPCR. We observed that mRNA expression levels of genes related to ion channels and Ca^2+^ handling were significantly upregulated in KCNJ2 OE iPSC-CMs as compared to Vector OE iPSC-CMs, including *SCN5A*, *CACNA1C*, *KCNH2* and *KCNQ1*, *ATP2A2* and *RYR2* (Fig. 6e). Moreover, KCNJ2 OE iPSC-CMs showed markedly upregulated mRNA expression of cardiac sarcomere-associated genes, including *TNNT2*, *ACTN2*, *MYBPC3*, *MYH7*, *MYH6* and *MYL7*, which was in close agreement to the RNA-Seq data (Fig. 6e). We also performed Western blot experiments to assess the protein expression of K_ir_2.1 (encoded by *KCNJ2*), Na_v_1.5 (encoded by *SCN5A*), RYR2 (encoded by *RYR2*), TNNT2 (encoded by *TNNT2*), MYBPC3 (encoded by *MYBPC3*), KCNH2 (encoded by *KCNH2*) and Ca_v_1.2 (encoded by *CACNA1C*) in Vector OE and KCNJ2 OE iPSC-CMs (Additional file 1: Figures 3–7). Significantly increased expression of K_ir_2.1 was detected in KCNJ2 OE iPSC-CMs as compared to Vector OE iPSC-CMs, indicating the successful overexpression of *KCNJ2* in iPSC-CMs (Additional file 1: Figure 3A–B). In line with our observations by RNA-Seq and qPCR, we observed that protein expression levels of Na_v_1.5, RYR2, TNNT2 and MYBPC3 were significantly increased in KCNJ2 OE iPSC-CMs, when compared to their Vector OE counterparts (Additional file 1: Fig. 3A, C–F). There was an increased trend of expression of KCNH2 and Ca_v_1.2 in KCNJ2 OE iPSC-CMs, but without statistical significance (Additional file 1: Figure 3A, G–H).

### Overexpression of *KCNJ2* enhances structural and metabolic maturation in iPSC-CMs

**Fig. 7:**
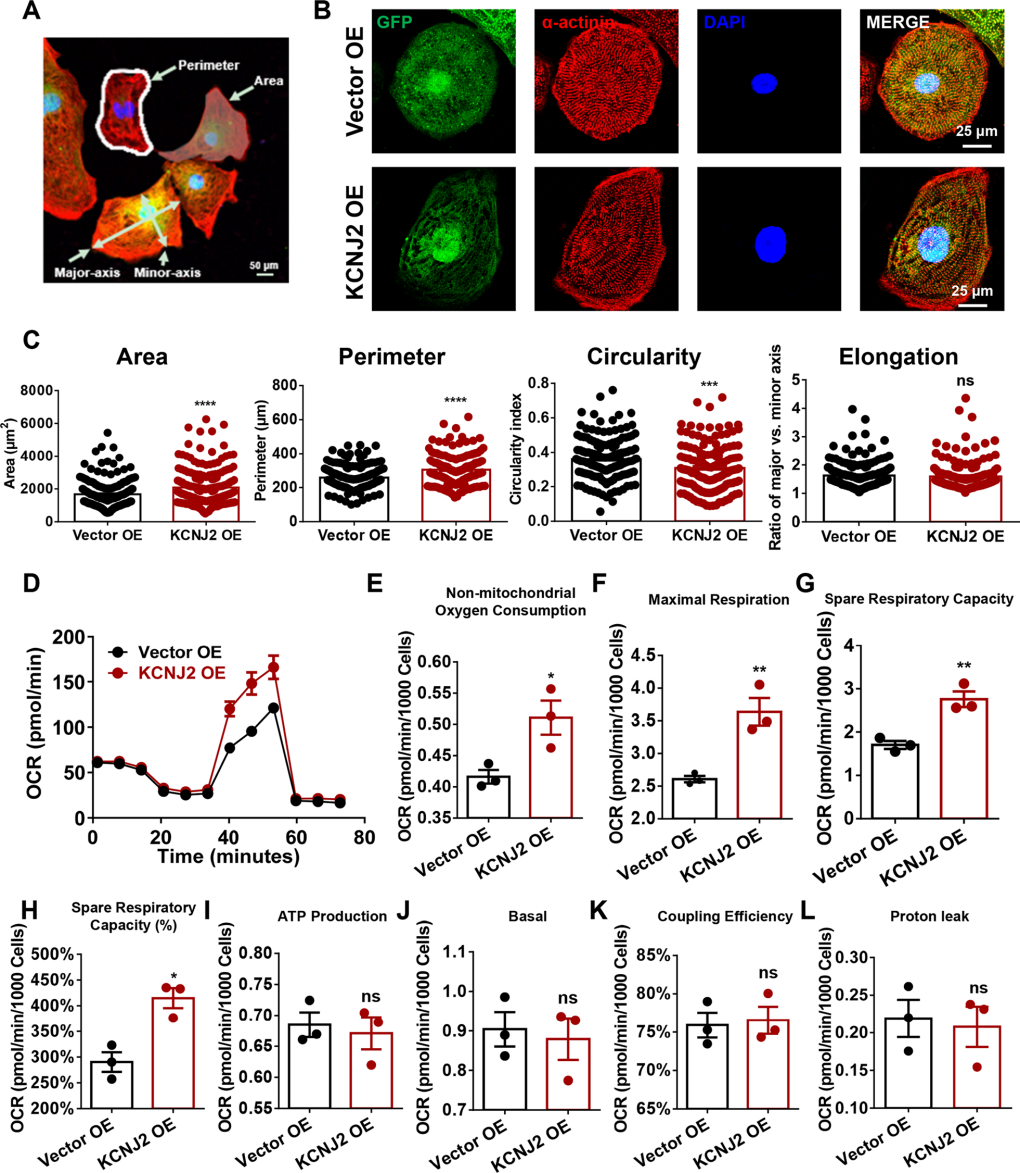
Overexpression of KCNJ2 enhances structural and metabolic maturation in iPSC-CMs. **a** Schematic diagram of morphological index analysis. **b** Representative confocal images of GFP (green) and α-actinin staining (red) in Vector OE and KCNJ2 OE iPSC-CMs. DAPI indicates nuclear staining (blue). Fluorescent detection was assessed with a confocal microscope (Nikon A1). Scale bar, 25 μm. **c** Bar graphs to compare the cell area, perimeter, circularity and elongation between Vector OE and KCNJ2 OE iPSC-CMs, respectively. Circularity is approximate to 4π × area × perimeter-2. Elongation is reflected with the ratio of major-axis length to minor-axis length. *n* = 148–203 cells. **d** The diagram depicts the trace of oxygen consumption rate (OCR) on Vector OE and KCNJ2 OE iPSC-CMs after sequentially administration of 1.5 μM oligomycin (ATP synthase inhibitor), 4 μM FCCP (uncoupler of oxidative phosphorylation in mitochondria) and 1 μM antimycin A (electron transport chain blocker), respectively. **e–l** Bar graphs to compare a series of fundamental parameters of mitochondrial function between Vector OE and KCNJ2 OE iPSC-CMs, including non-mitochondrial oxygen consumption, maximal respiration, spare respiratory capacity, spare respiratory capacity (%), ATP production, basal respiration, coupling efficiency and proton leak. *n* = 3 independent differentiations

To relate gene expression changes to functional consequences, we next assessed morphological characteristics of Vector OE and KCNJ2 OE iPSC-CMs by immunofluorescence with cardiac-specific marker α-actinin (Fig. 7a). The confocal imaging showed that Vector OE iPSC-CMs are round-shaped toward an immature cardiomyocyte morphology (Fig. 7b). In contrast, KCNJ2 OE iPSC-CMs displayed a more elongated and stretched shape, as evidenced by significantly enlarged cell area (Vector OE: 1671 ± 57.34 μm^2^; KCNJ2 OE: 2083 ± 75.62μm^2^), increased perimeter (Vector OE: 258.4 ± 5.976 μm; KCNJ2 OE: 304.2 ± 7.517 μm), and decreased circularity (Vector OE: 0.3612 ± 0.01004; KCNJ2 OE: 0.3085 ± 0.01105), pointing to a mature structural phenotype with more cylindrical morphology (Fig. 7b, c).

Given the distinct transcriptomic profile and the observed mature morphology in KCNJ2 OE iPSC-CMs, we hypothesized that the energy metabolism in these cells may be more active. We therefore functionally assessed the oxidative capacity in both Vector OE and KCNJ2 OE iPSC-CMs by Seahorse assay to measure oxygen consumption rate (OCR) (Fig. 7d). Interestingly, we observed that KCNJ2 OE iPSC-CMs showed significantly increased non-mitochondrial oxygen consumption (Vector OE: 0.4161 ± 0.01084; KCNJ2 OE: 0.5108 ± 0.02728), maximal respiration (Vector OE: 2.608 ± 0.04924; KCNJ2 OE: 3.638 ± 0.2107), and spare respiratory capacity (Vector OE: 1.704 ± 0.09104; KCNJ2 OE: 2.759 ± 0.1799), when compared to their Vector OE counterparts (Fig. 7e–l), suggesting that overexpression of *KCNJ2* can effectively enhance mitochondrial energy metabolism in iPSC-CMs.

### Preparation and evaluation of 3D human EHTs using KCNJ2 OE iPSC-CMs

**Fig. 8:**
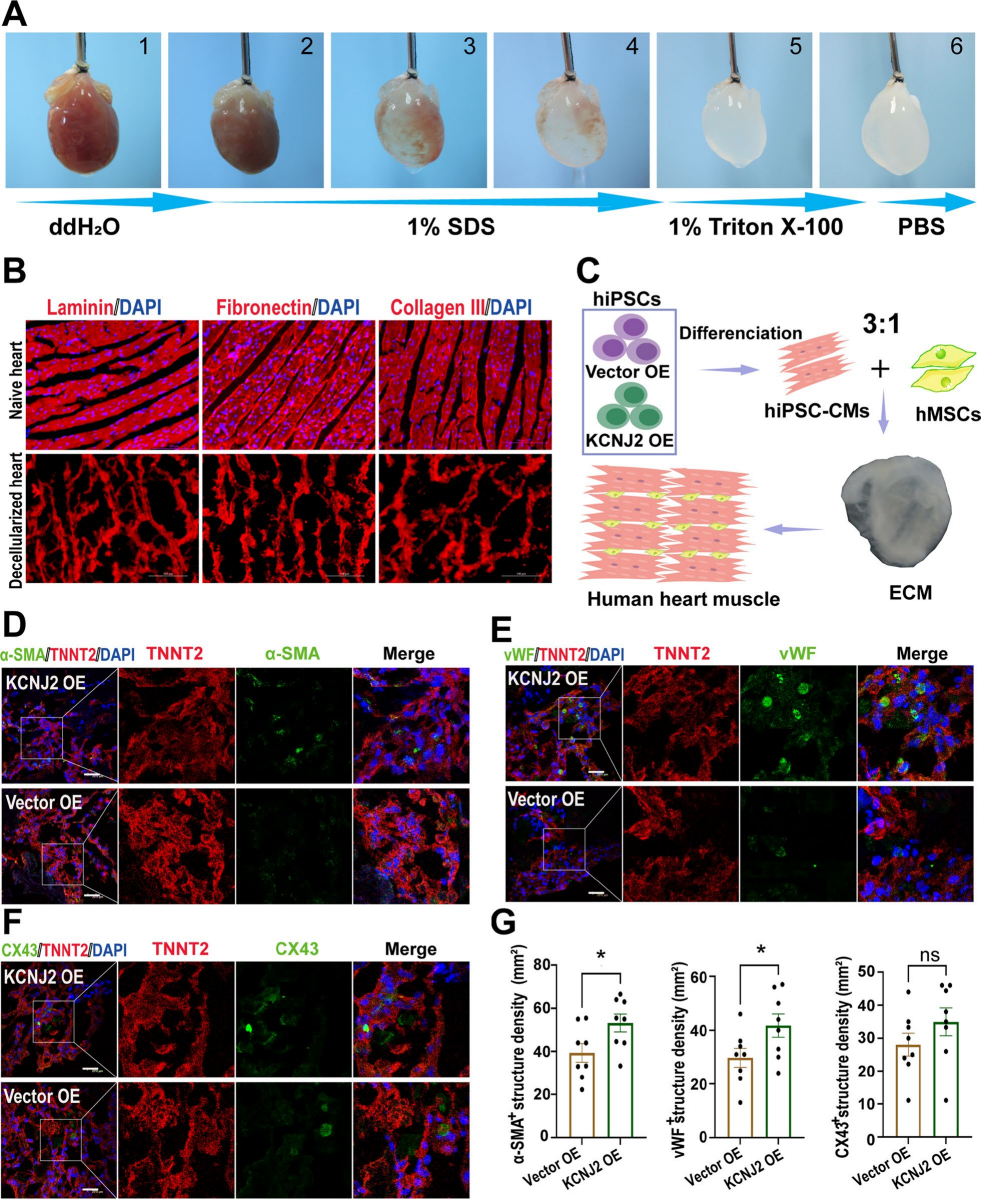
Preparation and evaluation of 3D human EHTs using KCNJ2 OE iPSC-CMs. **a** Process of decellularization of rat hearts and preparation of natural heart extracellular matrix (ECM). **b** Major ECM compositions (laminin, fibronectin and collagen III) of decellularized hearts and native rat hearts detected by immunofluorescence staining. Fluorescent detection was assessed with a confocal microscope (Leica TCS-SP8). Scale bar, 200 μm. **c** Schematic diagram of the preparation of 3D human engineered human heart tissues (EHTs) with or without KCNJ2 OE. This image was drawn by the authors. Double immunofluorescence staining for TNNT2/α-smooth muscle actin (α-SMA) (**d**), TNNT2/von Willebrand factor (vWF) (**e**), and TNNT2/connexin 43 (CX43) (**f**) in the EHTs. The right panels showed a higher magnification image of the boxed region in the left panel. Fluorescent detection was assessed with a confocal microscope (Leica TCS-SP8). Scale bars, 20 μm. **g** Quantification of the α-SMA+, vWF+ and CX43+ structures in the EHTs with or without KCNJ2 OE. *n* = 8 views.

We prepared decellularized natural heart ECM by continuous perfusion of isolated rat heart with detergents to remove the cellular components as previously described (Fig. 8a) [22]. Immunofluorescent staining indicated that DNA content of the decellularized ECM was nearly removed, and ECM compositions, such as laminin, fibronectin and collagen III, which remained present within the decellularized heart matrices (Fig. 8b). To mimic natural heart tissue, we used MSCs derived from human umbilical cord as the nonmyocyte component. In order to obtain optimal structural and functional properties, we combined 75% iPSC-CMs and 25% umbilical cord-derived nonmyocytes with pieces of decellularized natural rat heart ECM to generate 3D human EHTs (Fig. 8c). In comparison to Vector OE group, we observed significantly enhanced expression of α-SMA and vWF in the newly formed tissues of KCNJ2 OE EHTs by immunofluorescence, suggesting increased formation of smooth muscle and vascular endothelium, respectively (Fig. 8d–e, g). Meanwhile, in KCNJ2 OE EHTs, cardiac gap-junction protein CX43 was present at numerous points between adjacent cells, which promotes cell–cell contact and rapid electrical transmission for the heart tissues (Fig. 8f). We also observed an increased trend of CX43 expression in KCNJ2 OE EHTs (Fig. 8g). Collectively, these results demonstrate that co-culture of KCNJ2 OE iPSC-CMs and MSCs promotes the formation of vascular endothelium, smooth muscle and increases CX43 expression in 3D human EHTs, suggesting KCNJ2 OE iPSC-CMs could form mature human EHTs that possess better tissue structure and cell junction.

## Discussion

Here we performed a comprehensive characterization of morphological and functional changes in KCNJ2 OE iPSC-CMs to assess the effects of *KCNJ2* on the maturation of iPSC-CMs.iPSC-CMs have been widely used for modeling cardiac disease phenotypes and accurately evaluating the cardiotoxicity of potential therapeutic compounds, thus providing a novel tool that can bridge some of the gaps between animal-based models and adult human cardiomyocytes [7, 26, 27]. However, differences in ion-channel, Ca^2+^-handling and sarcomeric protein expression pattern suggest that iPSC-CMs are less mature than native adult cardiomyocytes. To this end, large efforts have been made to facilitate the maturation of iPSC-CMs by a wide variety of strategies, such as prolonged culture time [28, 29], hormone treatment [30–33], glucose replacement [12, 34, 35], substrate modification [36–38] and electrical stimulation [39].

The prominent immature phenotype is the spontaneous action potentials accompanied by the unstable and depolarized RMP in iPSC-CMs, which largely hampers their applications [40, 41]. The inwardly rectifying potassium channel K_ir_2.1, encoded by the *KCNJ2* gene, functions to stabilize the RMP and maintain the excitability in cardiomyocytes. Loss of K_ir_2.1-encoded I_K1_ is a major contributing factor to arrhythmogenesis in failing human hearts [42]. The low I_K1_ contributes to immature action potential that might bias drug-induced responses [14].

In comparison to adult cardiac LV tissues, we first found that the mRNA expression level of *KCNJ2* was much lower in iPSC-CMs. KCNJ2 OE iPSC-CMs yielded significantly enhanced protein expression of Kir2.1 and I_K1_. The enhanced I_K1_ linked to a quiescent phenotype that required pacing to elicit action potentials in KCNJ2 OE iPSC-CMs, which can be reversed by I_K1_ blocker BaCl_2_. Key parameters to shape the action potential morphology were also significantly changed in these cells, including hyperpolarized MDP, shortened APD and increased V_max_. Therefore, overexpression of *KCNJ2* to increase I_K1_ robustly enhances electrophysiological maturation in iPSC-CMs.

KCNJ2 OE iPSC-CMs exhibited a distinct transcriptomic profile. The upregulated mRNA expression level of *SCN5A* suggests larger Na^+^ currents that are in line with the accelerated depolarization. *CACNA1C*, *KCNH2* and *KCNQ1* encode L-type Ca^2+^ currents and delayed rectifier K^+^ currents I_Kr_ and I_Ks_, respectively, which play important role in the repolarization of phase 2 and 3 in cardiomyocytes. Upregulated mRNA expression levels of 3 forementioned genes may contribute to the prominent plateau phase of action potentials observed in KCNJ2 OE iPSC-CMs. In addition, *ATP2A2* encodes SERCA2a that mediates Ca^2+^ in the cytoplasm to re-enter into the SR to maintain a low cytoplasmic concentration of Ca^2+^ ions, and *RYR2* encodes RYR2 that mediates the release of Ca^2+^ from the SR into the cytoplasm and thereby plays a key role in triggering cardiac muscle contraction. The upregulation of *ATP2A2* and *RYR2* mRNA expression in KCNJ2 OE iPSC-CMs was in agreement to the improved Ca^2+^ signaling.

An observation not previously described was the cardiomyocyte morphological change by KCNJ2 OE. As compared to Vector OE counterparts, KCNJ2 OE iPSC-CMs displayed enlarged cell size and more elongated and stretched shape, indicating a morphological phenotype toward structural maturation. This finding was consistent with our RNA-Seq data in which mRNA expression levels of a series of cardiac sarcomere-associated genes were upregulated. Moreover, KCNJ2 OE iPSC-CMs showed a significant improvement in OCR parameters, suggesting that KCNJ2 plays a pivotal role in metabolic maturation of iPSC-CMs.

It has been reported that due to the absence of I_K1_, MDP of iPSC-CMs depends critically on I_Kr_ [43]. Consistently, because of I_Kr_ blockade, treatment of E-4031 largely affected the MDP in Vector OE iPSC-CMs, resulting in depolarized MDP, especially at high concentrations. However, given sufficient I_K1_, KCNJ2 OE iPSC-CMs maintained more stable MDP when treated with E-4031. As a consequence, E-4031-induced APD prolongation can be observed in KCNJ2 OE iPSC-CMs in a concentration-dependent manner, but not in Vector OE iPSC-CMs. Our findings support that use of electrophysiological mature iPSC-CMs may provide more accurate prediction of drug-induced cardiotoxicity, especially for hERG blockers.

Tissue engineering is an emerging interdisciplinary that integrates biology with engineering to manufacture functional tissue, such as engineered myocardial tissue [44]. Generation of 3D human EHTs using iPSC-CMs can mimic intricate architecture in the native myocardium by promoting anisotropic cellular alignment and synergistic electromechanical interactions [44]. We found that, via the strategy of *KCNJ2* overexpression in iPSC-CMs, the generated human EHTs showed increased formation of smooth muscle, vascular endothelium and gap junction, which can provide a more mature tissue-level model for cardiovascular research.

It is important to note that there are several limitations in our study. First, overexpression of *KCNJ2* in iPSC-CMs causes a distinct transcriptomic profile, in which multiple genes encoding ion channels, Ca^2+^ handling proteins and cardiac sarcomeric proteins are significantly upregulated. Since cardiomyocytes developed in vivo are regulated by various factors, combined strategies might better promote the maturation of iPSC-CMs. Second, we show that overexpression of *KCNJ2* can robustly enhance maturation of iPSC-CMs. However, there is currently a lack of criteria for the maturation of iPSC-CMs in the field. Third, the accurate mechanism of *KCNJ2* overexpression promoting maturation of iPSC-CMs needs to be further elucidated. Finally, no in vivo experiments were performed in this study. It has been demonstrated that iPSC-CMs with a higher degree of maturation are capable of preserving cardiac function and tissue viability when transplanted into a mouse model of acute myocardial infarction (AMI) [45]. In future studies, it is therefore worth exploring whether iPSC-CMs overexpressing *KCNJ2* may have a better repair effect on AMI in animal models.

## Conclusions

Overexpression of *KCNJ2* can robustly enhance maturation of iPSC-CMs in electrophysiology, Ca^2+^ signaling, metabolism, transcriptomic profile, cardiomyocyte structure and tissue engineering, thus providing more accurate cellular model for elucidating cellular and molecular mechanisms of cardiovascular diseases, screening drug-induced cardiotoxicity, and developing personalized and precision cardiovascular medicine (Additional file 1: Figure 8).

## Supplementary Information

**Additional file 1. Supplemental Figure 1**. Characterization of iPSC#5 and iPSC#6. **Supplemental Figure 2**. Full length blots of K_ir_2.1 expression in untreated, Vector OE and KCNJ2 OE iPSC-CMs. **Supplemental Figure 3**. Expression of a panel of seven proteins in Vector OE and KCNJ2 OE iPSC-CMs. **Supplemental Figure 4**. Full length blots of K_ir_2.1 and Nav1.5 expression in Vector OE and KCNJ2 OE iPSC-CMs. **Supplemental Figure 5**. Full length blots of RYR2 and TNNT2 expression in Vector OE and KCNJ2 OE iPSC-CMs. **Supplemental Figure 6**. Full length blots of MYBPC3 and KCNH2 expression in Vector OE and KCNJ2 OE iPSC-CMs. **Supplemental Figure 7**. Full length blots of Cav1.2 and GAPDH expression in Vector OE and KCNJ2 OE iPSC-CMs. **Supplemental Figure 8**. Schematic representation of the approach to enhance maturation by overexpressing KCNJ2 in iPSC-CMs, which can provide more accurate prediction of drug-induced cardiotoxicity, and form more mature 3D human engineered heart tissues with better tissue structure and cell junction. **Supplemental Table 1**. Clinical features of six recruited patients. **Supplemental Table 2**. Summary of iPSC lines in this study. **Supplemental Table 3**. Primers used for qPCR in this study.

## Data Availability

The accession number for the RNA-Seq data reported in this study is PRJNA837990 (name of database: studies of iPS-CMs overexpressing KCNJ2). The data that support our findings of this study are available from the corresponding author on reasonable request.

## Abbreviations

iPSCs: Induced pluripotent stem cells
iPSC-CMs: IPSC-derived cardiomyocytes
RMP: Resting membrane potential
SR: Sarcoplasmic reticulum
LV: Left ventricular
ALP: Alkaline phosphatase
FACS: Fluorescence-activated cell sorting
EHTs: Engineered heart tissues
ECM: Extracellular matrix
VWF: Von Willebrand Factor
CX43: Connexin 43
MDP: Maximal diastolic potential
APA: Action potential amplitude
APD_50_: Action potential duration at 50%
APD_90_: Action potential duration at 90%
V_max_: Maximal upstroke velocity
qPCR: Real-time quantitative PCR
MSCs: Mesenchymal stem cells
