# Supplementary material for: Overexpression of KCNJ2 enhances maturation of human-induced pluripotent stem cell-derived cardiomyocytes
Source: Stem Cell Res Ther. 2023 Apr 15;14:92. doi: 10.1186/s13287-023-03312-9 (PMC10105952; doi:10.1186/s13287-023-03312-9)
Supplement: Supplementary file 1 — Additional file 1. Supplemental Figure 1. Characterization of iPSC#5 and iPSC#6. Supplemental Figure 2. Full length blots of Kir2.1 expression in untreated, Vector OE and KCNJ2 OE iPSC-CMs. Supplemental Figure 3. Expression of a panel of seven proteins in Vector OE and KCNJ2 OE iPSC-CMs. Supplemental Figure 4. Full length blots of Kir2.1 and Nav1.5 expression in Vector OE and KCNJ2 OE iPSC-CMs. Supplemental Figure 5. Full length blots of RYR2 and TNNT2 expression in Vector OE and KCNJ2 OE iPSC-CMs. Supplemental Figure 6. Full length blots of MYBPC3 and KCNH2 expression in Vector OE and KCNJ2 OE iPSC-CMs. Supplemental Figure 7. Full length blots of Cav1.2 and GAPDH expression in Vector OE and KCNJ2 OE iPSC-CMs. Supplemental Figure 8. Schematic representation of the approach to enhance maturation by overexpressing KCNJ2 in iPSC-CMs, which can provide more accurate prediction of drug-induced cardiotoxicity, and form more mature 3D human engineered heart tissues with better tissue structure and cell junction. Supplemental Table 1. Clinical features of six recruited patients. Supplemental Table 2. Summary of iPSC lines in this study. Supplemental Table 3. Primers used for qPCR in this study. [file 13287_2023_3312_MOESM1_ESM.pdf]

Additional file 1: Supporting information

Overexpression of *KCNJ2* enhances maturation of human induced pluripotent stem cell-derived cardiomyocytes

Jingjun Zhou[1, 2#], Baiping Cui[3, 4#], Xiaochen Wang[1, 2#], Hongkun Wang[1, 2], Junnan Zheng[5], Fengfeng Guo[1, 2], Yaxun Sun[6], Hangping Fan[1, 2], Jiaxi Shen[1, 2], Jun Su[1, 2], Jue Wang[1, 2], Haige Zhao[5], Yiquan Tang[7], Tingyu Gong[1, 2*], Ning Sun[8*], Ping Liang[1, 2*]

[1]Key Laboratory of combined Multi-organ Transplantation, Ministry of Public Health, the First Affiliated Hospital, Zhejiang University School of Medicine, Hangzhou, Zhejiang 310003, China

[2]Institute of Translational Medicine, Zhejiang University, Hangzhou, Zhejiang 310029, China

[3]Institute of Geriatrics (Shanghai University), Affiliated Nantong Hospital of Shanghai University (The Sixth People's Hospital of Nantong), School of Medicine, Shanghai University, Nantong, 226011, China

[4]Shanghai Engineering Research Center of Organ Repair, School of Medicine, Shanghai University, Shanghai 200444, China

[5]Department of Cardiovascular Surgery, the First Affiliated Hospital, Zhejiang University School of Medicine, Hangzhou, Zhejiang 310003, China

[6]Department of Cardiology, Sir Run Run Shaw Hospital, Zhejiang University School of Medicine, Hangzhou, Zhejiang 310016, China

[7]State Key Laboratory of Medical Neurobiology and MOE Frontiers Center for Brain Science, Institutes of Brain Science, Fudan University, Shanghai 200032, China

[8]Wuxi School of Medicine, Jiangnan University, Wuxi, Jiangsu 214028, China

[#]These authors contributed equally to this work

[*]**Correspondence:** Ping Liang, MD, PhD, 79 Qingchun Road, Hangzhou, Zhejiang 310003, China. Phone: 86-571-86971872, E-mail: pingliang@zju.edu.cn; Ning Sun, MD, PhD, 1800 Lihu Avenue, Wuxi, Jiangsu 214028, China. E-mail: sunning@jiangnan.edu.cn; Tingyu Gong, MD, PhD, 79 Qingchun Road, Hangzhou, Zhejiang 310003, China. E-mail: 11918392@zju.edu.cn

List of Supporting information

Supplemental Figures

- **Supplemental Figure 1.** Characterization of iPSC#5 and iPSC#6.
- **Supplemental Figure 2.** Full length blots of $K_{ir}2.1$ expression in untreated, Vector OE and KCNJ2 OE iPSC-CMs.
- **Supplemental Figure 3.** Expression of a panel of seven proteins in Vector OE and KCNJ2 OE iPSC-CMs.
- **Supplemental Figure 4.** Full length blots of $K_{ir}2.1$ and $Na_v1.5$ expression in Vector OE and KCNJ2 OE iPSC-CMs.
- **Supplemental Figure 5.** Full length blots of RYR2 and TNNT2 expression in Vector OE and KCNJ2 OE iPSC-CMs.
- **Supplemental Figure 6.** Full length blots of MYBPC3 and KCNH2 expression in Vector OE and KCNJ2 OE iPSC-CMs.
- **Supplemental Figure 7.** Full length blots of Cav1.2 and GAPDH expression in Vector OE and KCNJ2 OE iPSC-CMs.
- **Supplemental Figure 8.** Schematic representation of the approach to enhance maturation by overexpressing *KCNJ2* in iPSC-CMs, which can provide more accurate prediction of drug-induced cardiotoxicity, and form more mature 3D human engineered heart tissues with better tissue structure and cell junction.

Supplemental Tables

- **Supplemental Table 1.** Clinical features of six recruited patients.
- **Supplemental Table 2.** Summary of iPSC lines in this study.
- **Supplemental Table 3.** Primers used for qPCR in this study.

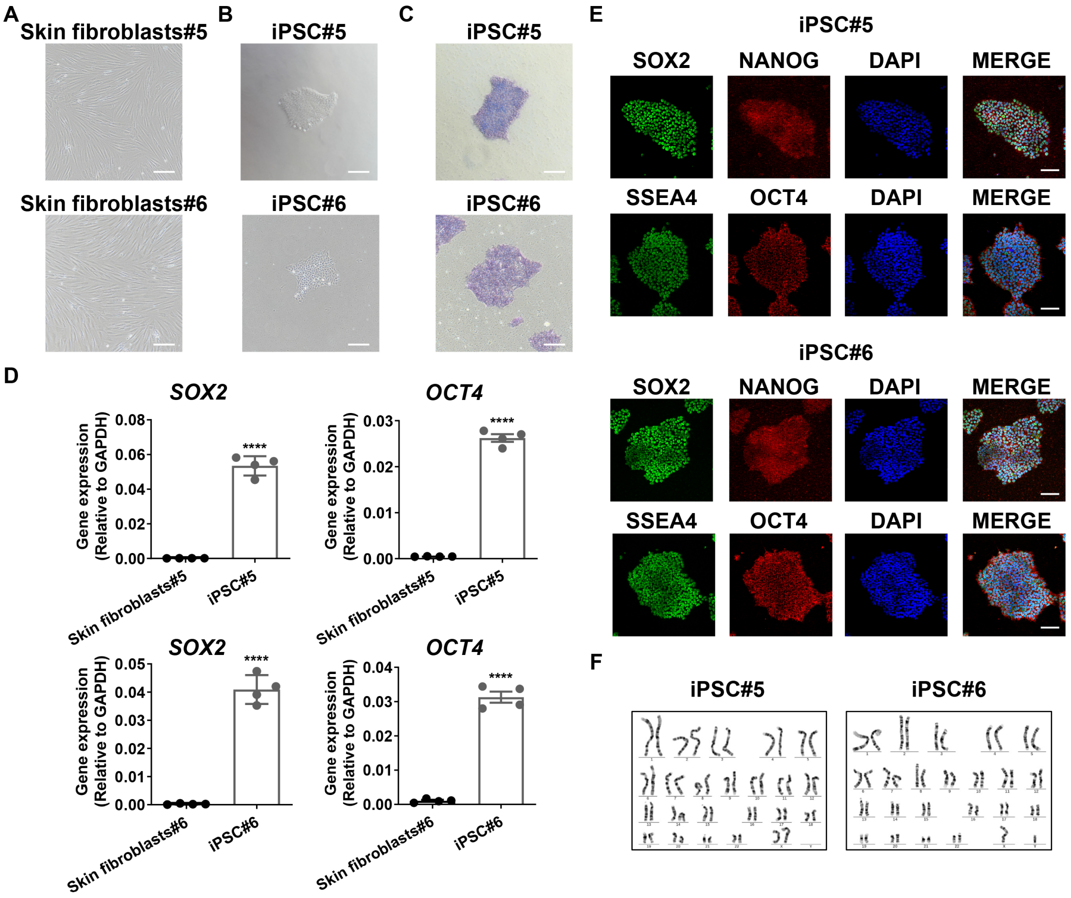

Supplemental Figure 1. Characterization of iPSC#5 and iPSC#6. A. Typical morphology of skin fibroblasts. The images were captured by an inverted fluorescence microscope (Nikon ECLIPSE Ti-S). Scale bar, 100 µm. **B.** Typical morphology of iPSCs. The images were captured by an inverted fluorescence microscope (Nikon ECLIPSE Ti-S). Scale bar, 100 µm. **C.** Representative graphs of ALP staining of iPSCs. The images were captured by an inverted fluorescence microscope (Nikon ECLIPSE Ti-S). Scale bar, 100 µm. **D.** Bar graphs to compare the mRNA expression of *SOX2* and *OCT4* by qPCR between skin fibroblasts and iPSCs. n= 4. **E.** Representative confocal images of pluripotent staining of iPSCs using SOX2 (green), NANOG (red), SSEA4 (green) and OCT4 (red), DAPI indicates nuclear staining (blue). Fluorescent detection was assessed with a confocal microscope (Nikon A1). Scale bar, 100 µm. **F.** Representative graphs of karyotypes of iPSCs.

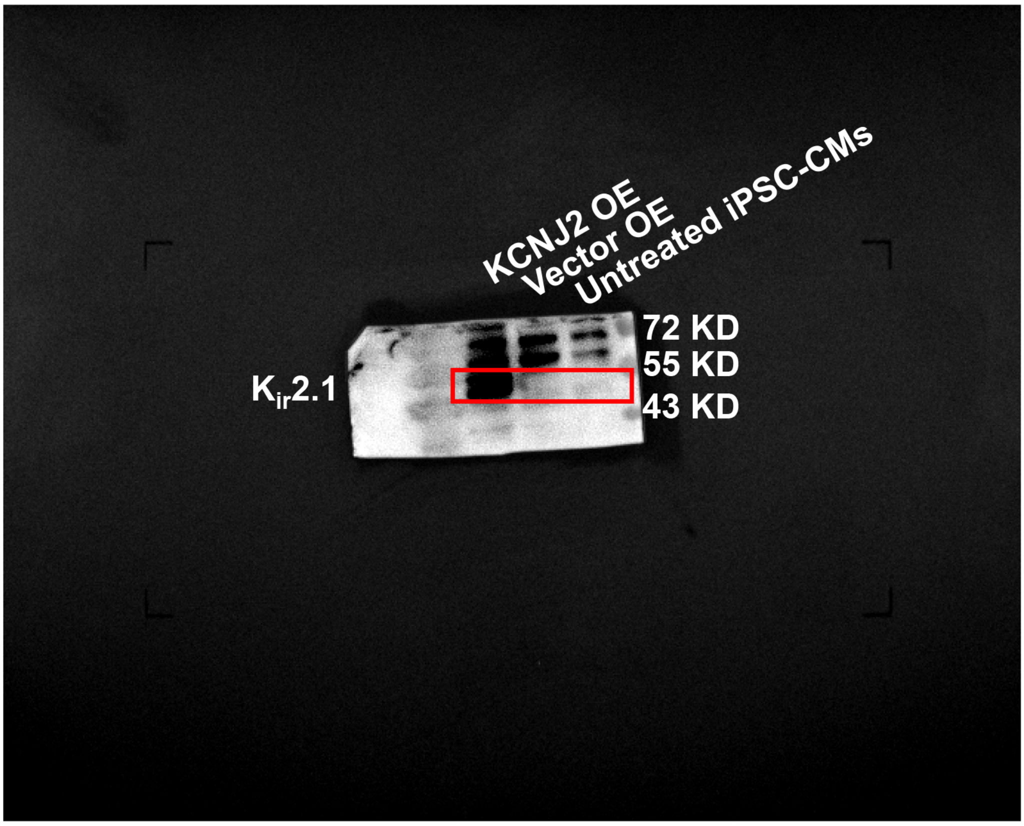

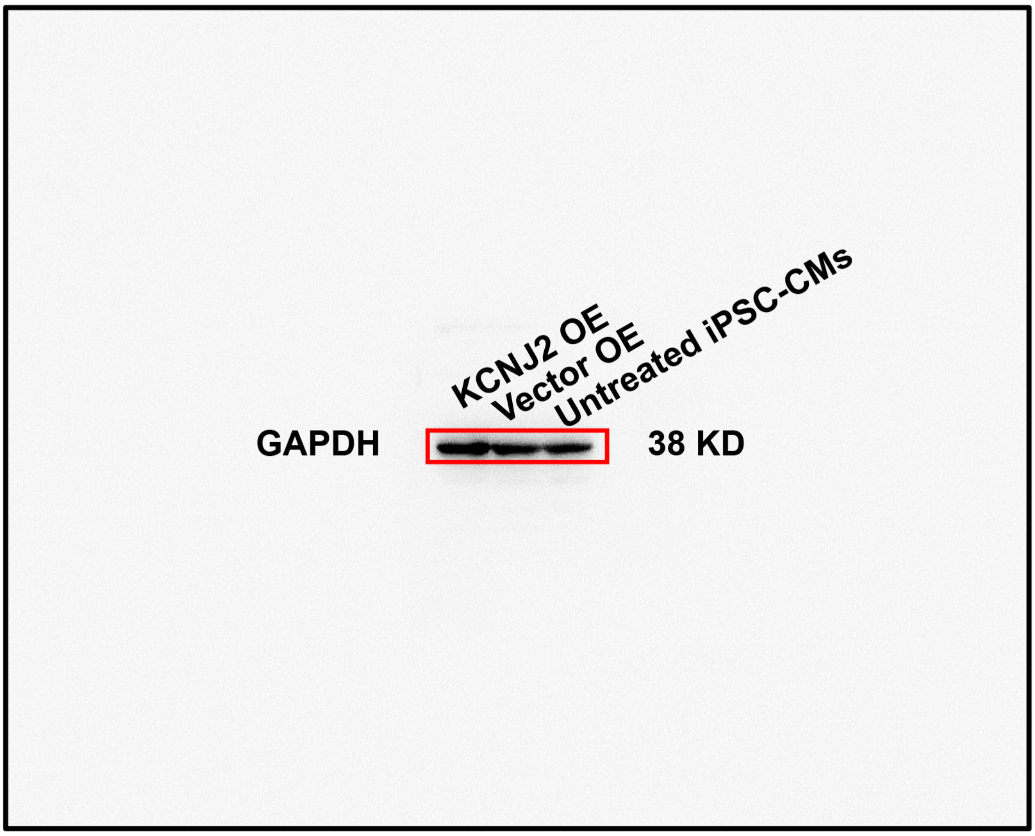

Supplemental Figure 2. Full length blots of K_{ir}2.1 expression in untreated, Vector OE and KCNJ2 OE iPSC-CMs. Red boxes indicate the cropped blots shown in Figure 2C.

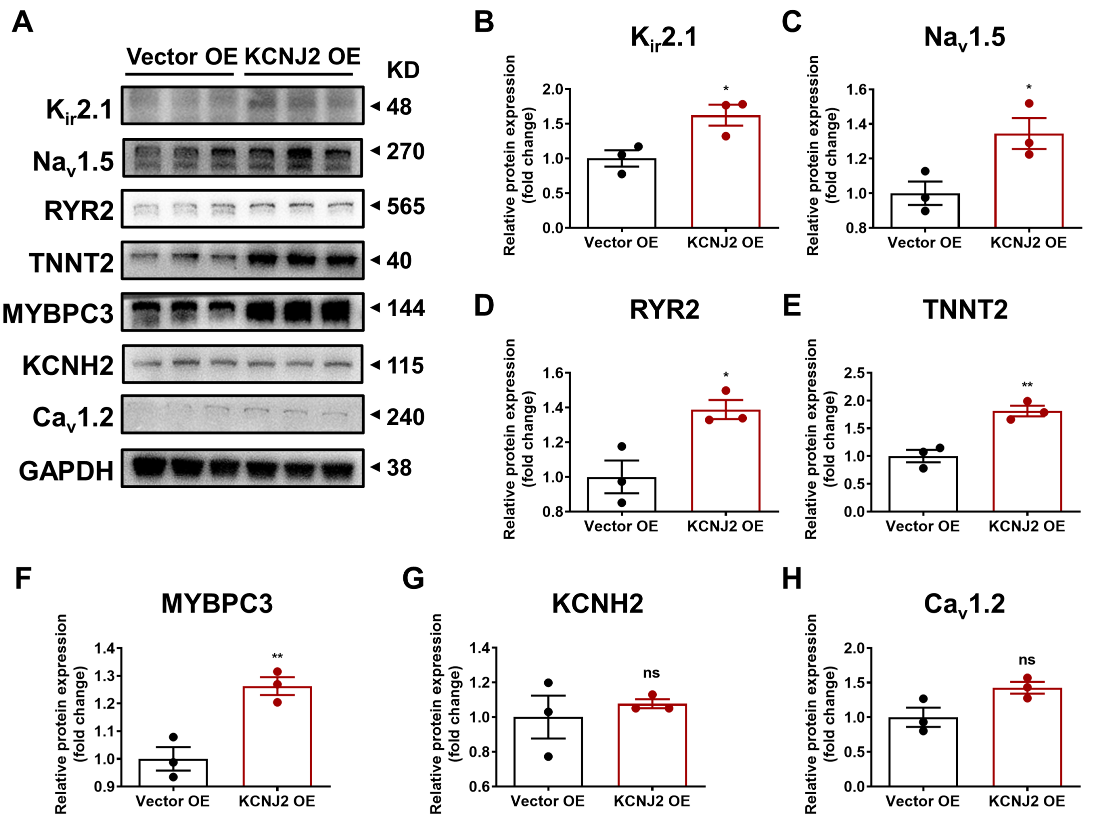

Supplemental Figure 3. Expression of a panel of seven proteins in Vector OE and KCNJ2 OE iPSC-CMs. A. Western blot analysis of a panel of seven proteins in Vector OE and KCNJ2 OE iPSC-CMs, including ion channels, Ca^{2+} handling proteins, and cardiac sarcomere proteins. Full-length blots are presented in Supplemental Figure 4-7. **B-H.** Bar graphs to compare the protein expression of $K_{ir}2.1$, $Na_V1.5$, RYR2, TNNT2, MYBPC3, KCNH2 and $Ca_V1.2$ between Vector OE and KCNJ2 OE iPSC-CMs. n= 3 independent differentiations.

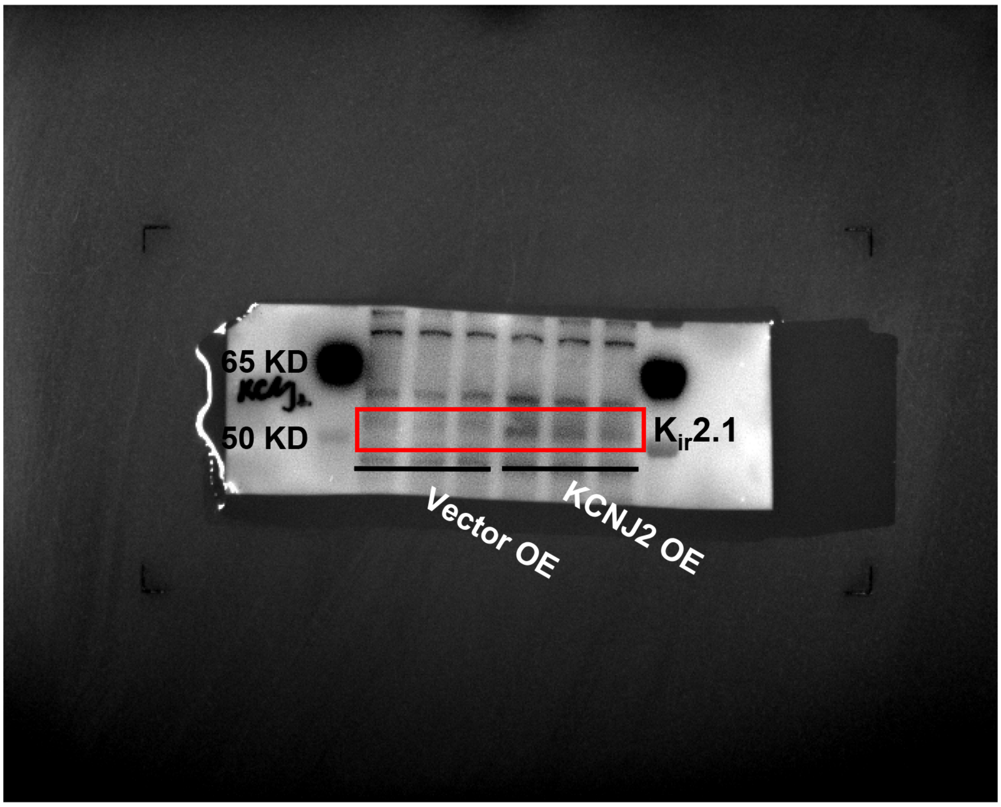

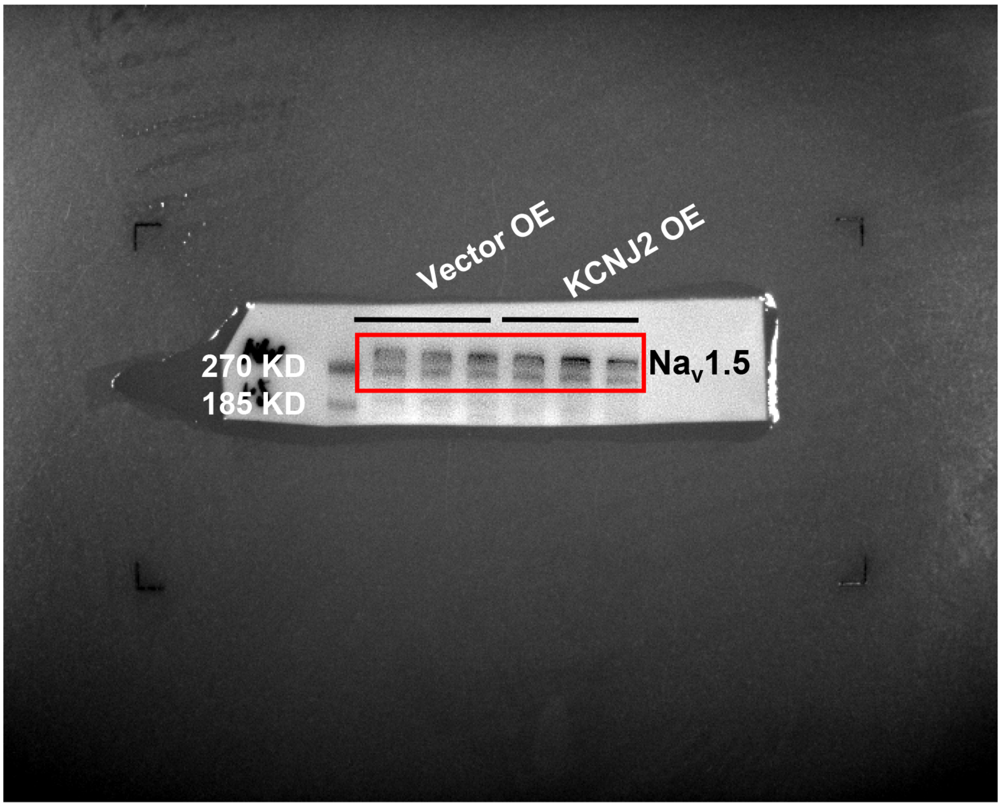

Supplemental Figure 4. Full length blots of $K_{ir}2.1$ and $Na_V1.5$ expression in Vector OE and KCNJ2 OE iPSC-CMs. Red boxes indicate the cropped blots shown in Supplemental Figure 3A.

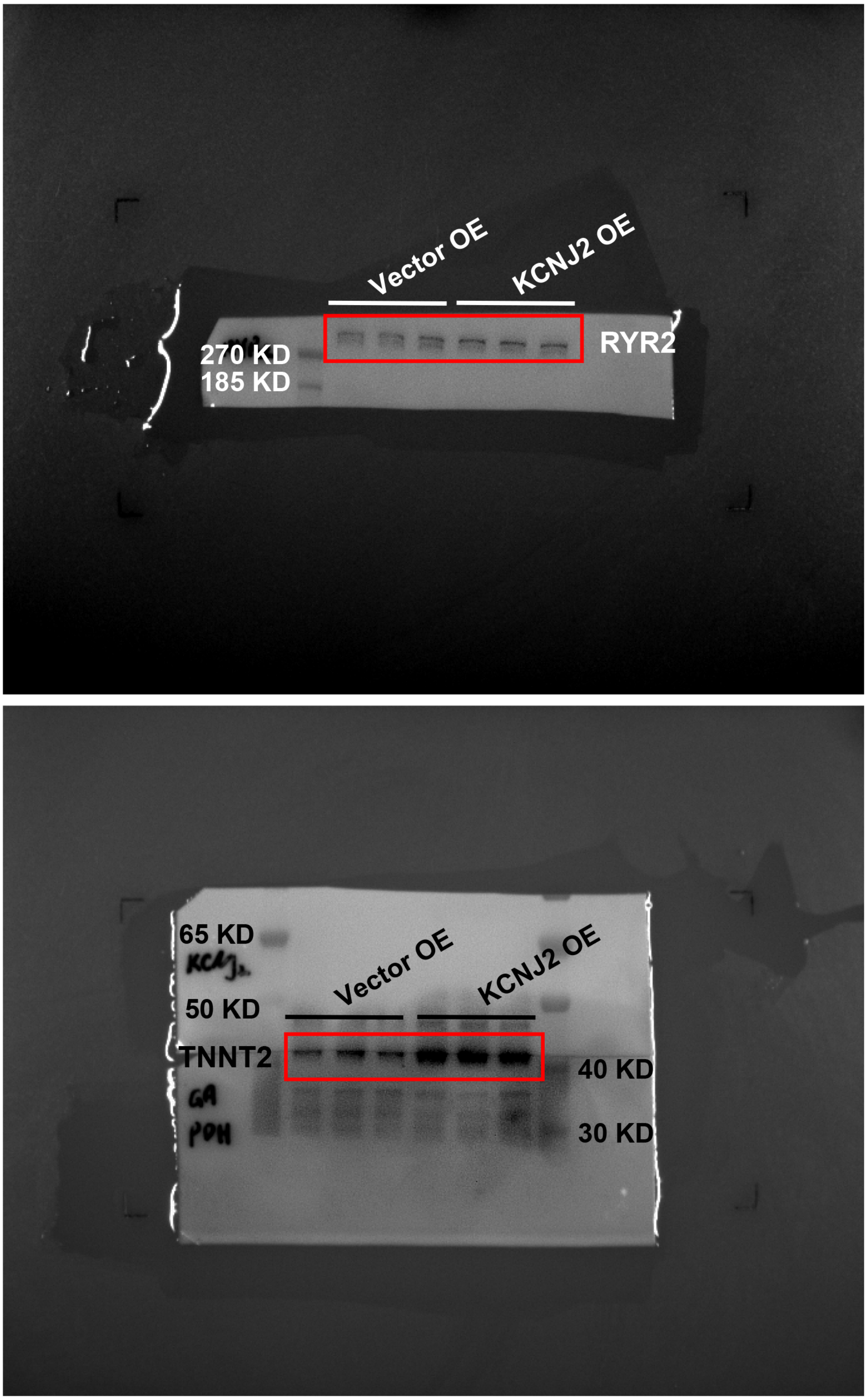

Supplemental Figure 5. Full length blots of RYR2 and TNNT2 expression in Vector OE and KCNJ2 OE iPSC-CMs. Red boxes indicate the cropped blots shown in Supplemental Figure 3A.

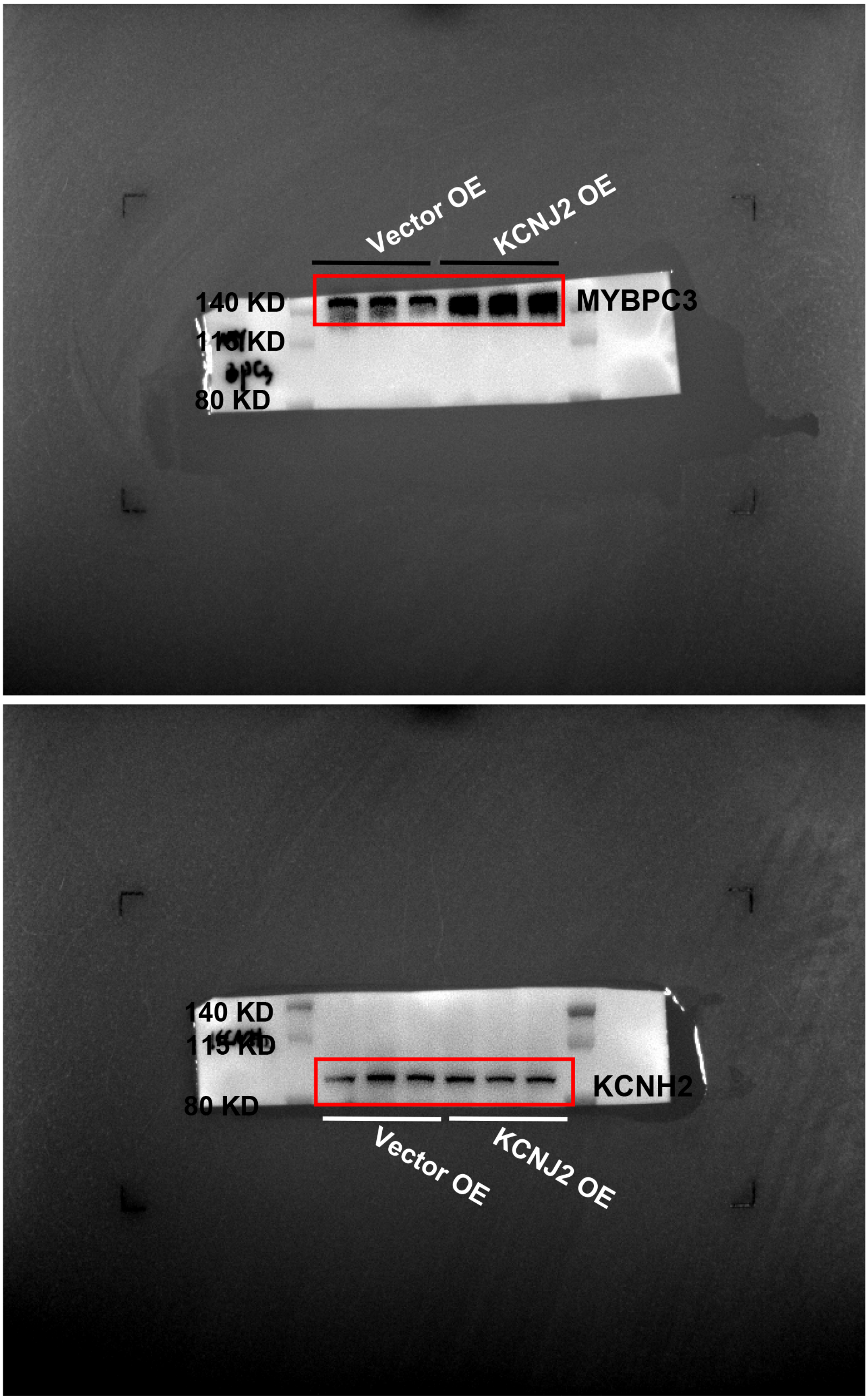

Supplemental Figure 6. Full length blots of MYBPC3 and KCNH2 expression in Vector OE and KCNJ2 OE iPSC-CMs. Red boxes indicate the cropped blots shown in Supplemental Figure 3A.

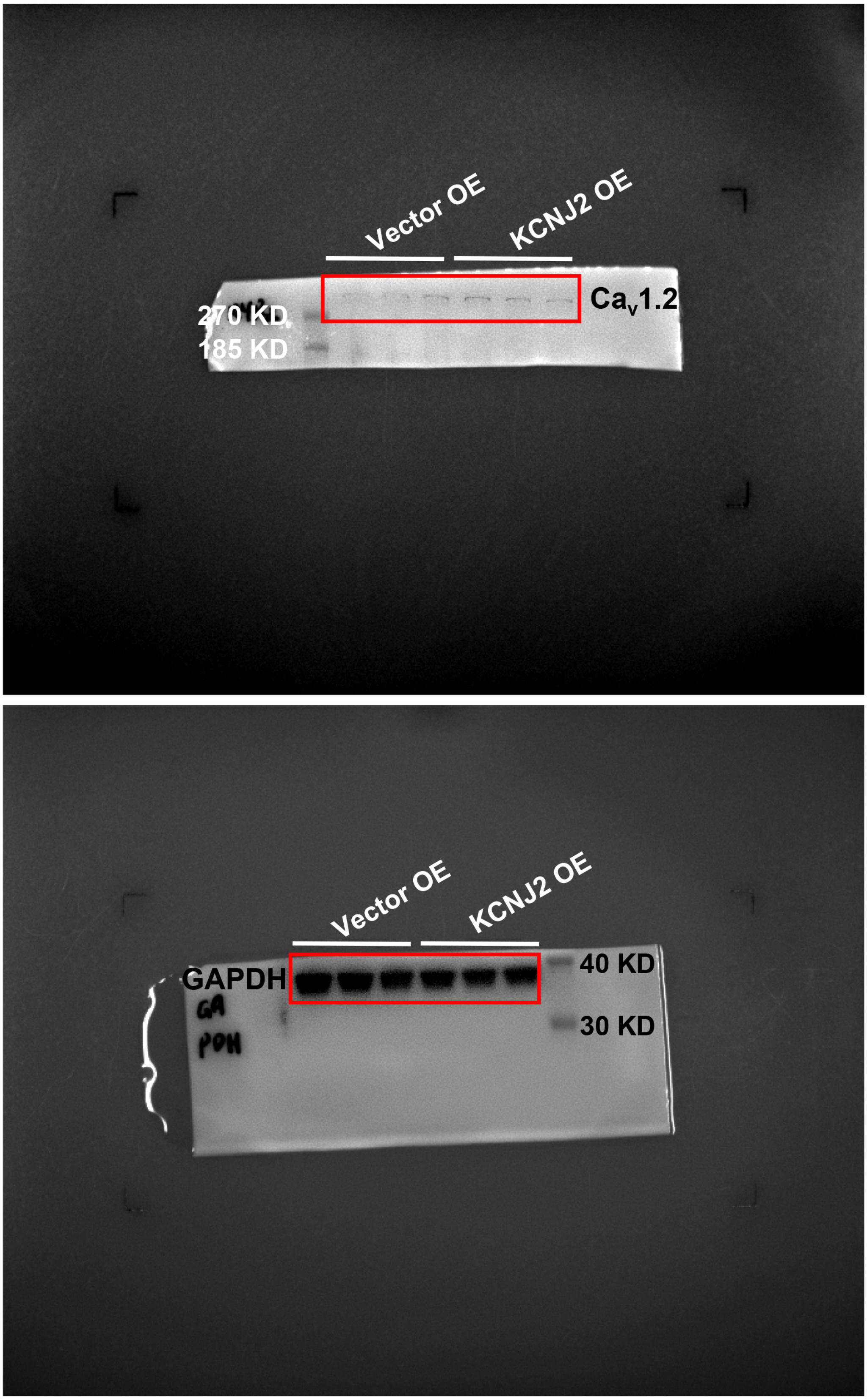

Supplemental Figure 7. Full length blots of $Ca_V1.2$ and GAPDH expression in Vector OE and KCNJ2 OE iPSC-CMs. Red boxes indicate the cropped blots shown in Supplemental Figure 3A.

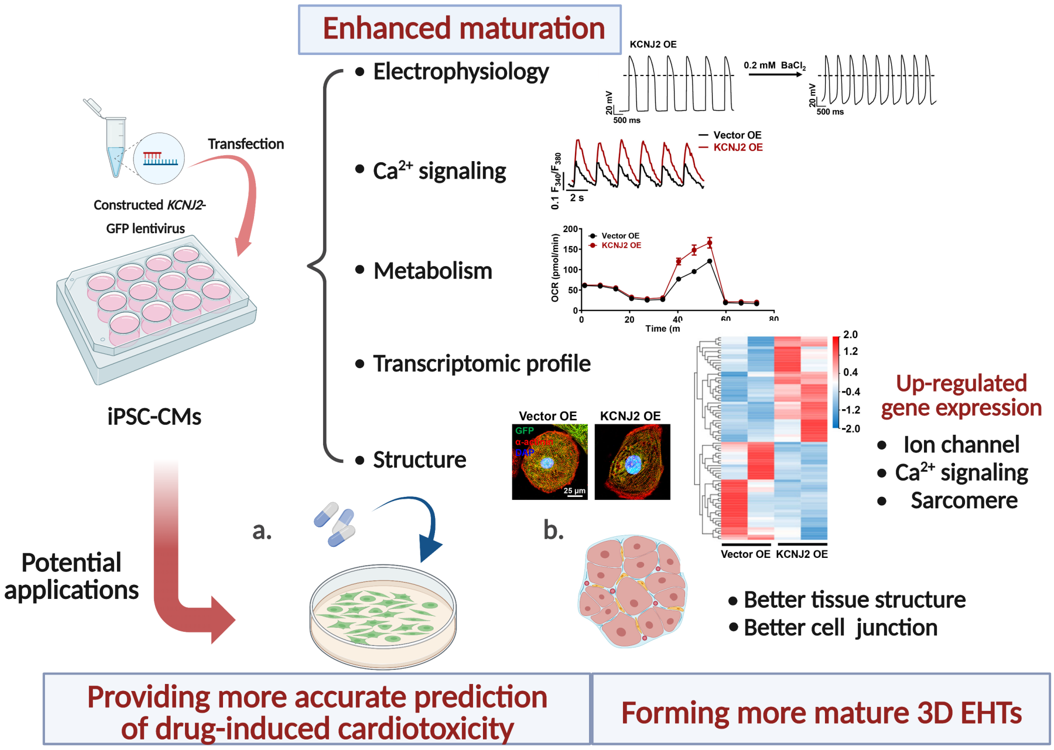

Supplemental Figure 8. Schematic representation of the approach to enhance maturation by overexpressing *KCNJ2* in iPSC-CMs, which can provide more accurate prediction of drug-induced cardiotoxicity, and form more mature 3D human engineered heart tissues with better tissue structure and cell junction. This image was drawn by the authors (created with BioRender.com).

Supplemental Table 1. Clinical features of six recruited patients

Patient ID	Age (years)	Sex	Valvular disease
**Patient 1**	30-39	1	Mitral stenosis, aortic regurgitation
**Patient 2**	60-69	1	Aortic regurgitation
**Patient 3**	50-59	2	Aortic stenosis with aortic regurgitation
**Patient 4**	60-69	1	Mitral stenosis with regurgitation, aortic stenosis with regurgitation
**Patient 5**	70-79	2	Aortic regurgitation
**Patient 6**	50-59	1	Mitral stenosis with regurgitation, aortic stenosis with regurgitation

Supplemental Table 2. Summary of iPSC lines in this study

	Sex	Ethnicity	Somatic cells	Reprogramming method	References
**iPSC#1**	2	Han Chinese	Skin fibroblasts	Sendai virus	*J Mol Cell Cardiol* 2020;142:53-64.
**iPSC#2**	1	Han Chinese	Skin fibroblasts	Sendai virus	*J Mol Cell Cardiol* 2020;142:53-64.
**iPSC#3**	1	Han Chinese	Skin fibroblasts	Sendai virus	*Clin Transl Med* 2021;11(9):e549.
**iPSC#4**	1	Han Chinese	Skin fibroblasts	Sendai virus	*Clin Transl Med* 2021;11(9):e549.
**iPSC#5**	1	Han Chinese	Skin fibroblasts	Sendai virus	Unpublished
**iPSC#6**	2	Han Chinese	Skin fibroblasts	Sendai virus	Unpublished

Supplemental Table 3. Primers used for qPCR in this study

Genes	Primer sequences		Gene Description
SOX2	Forward	CCCAGCAGACTTCACATGT	SRY-box transcription factor 2
	Reverse	CCTCCCATTTCCCTCGTTTT	
OCT4	Forward	CCTCACTTCACTGCACTGTA	Organic cation/carnitine transporter4
	Reverse	CAGGTTTTCTTTCCCTAGCT	
TNNT2	Forward	GGAGGAGTCCAAACCAAAGCC	Troponin T2, cardiac type
	Reverse	TCAAAGTCCACTCTCTCTCCATC	
ACTN2	Forward	CAAACCTGACCGGGGAAAAT	Actinin alpha 2
	Reverse	CTGAATAGCAAAGCGAAGGATGA	
MYBPC3	Forward	GGCATGCTAAAGAGGCTCAA	Myosin binding protein C3
	Reverse	TCTTGTGGCCTTTGCTCAC	
MYH7	Forward	ACTGCCGAGACCGAGTATG	Myosin heavy chain 7
	Reverse	GCGATCCTTGAGGTTGTAGAGC	
MYH6	Forward	GCTGGTCACCAACAATCCCTA	Myosin heavy chain 6
	Reverse	CGTCAAAGGCACTATCGGTGG	
MYL7	Forward	GCCCAACGTGGTTCTTCCAA	Myosin light chain 7

	Reverse	CTCCTCCTCTGGGACACTC	
ATP2A2	Forward	TACTTCTGTTATCTGCTCAGAC	Sarcoplasmic/endoplasmic reticulum Ca^{2+} transporting 1
	Reverse	CTGTCCAGAATGAACATCCT	
RYR2	Forward	CAGGACAGGAATCTTATGTCTG	Ryanodine receptor 2
	Reverse	CTGTTTCCGGAAGCAATCC	
SCN5A	Forward	TCACCGCCATTTACACCTTTG	Sodium voltage-gated channel alpha subunit 5
	Reverse	GGTCCCGAAGGAAAGTGAACG	
CACNA1C	Forward	AATCGCCTATGGACTCCTCTT	Calcium voltage-gated channel subunit alpha1 C
	Reverse	GCGCCTTCACATCAAATCCG	
KCNH2	Forward	CACCGCCCTGTACTTCATCT	Potassium voltage-gated channel subfamily H member 2
	Reverse	AGGCCTTGCATACAGGTTCA	
KCNQ1	Forward	CGCCTGAACCGAGTAGAAGA	Potassium voltage-gated channel subfamily Q member 1
	Reverse	TGAAGCATGTCGGTGATGAG	
KCNJ2	Forward	GTGCGAACCAACCGCTACA	Potassium voltage-gated channel subfamily J member 2
	Reverse	CCAGCGAATGTCCACACAC	
GAPDH	Forward	GGTCGGAGTCAACGGATTTG	Glyceraldehyde-3-phosphate dehydrogenase
	Reverse	CGGTGCCATGGAATTTGCC	
